# The potential for a CRISPR gene drive to eradicate or suppress globally invasive social wasps

**DOI:** 10.1038/s41598-020-69259-6

**Published:** 2020-07-24

**Authors:** Philip J. Lester, Mariana Bulgarella, James W. Baty, Peter K. Dearden, Joseph Guhlin, John M. Kean

**Affiliations:** 1 School of Biological Sciences, Victoria University of Wellington, PO Box 600, Wellington, New Zealand; 2 Genomics Aotearoa and Biochemistry Department, University of Otago, Dunedin, New Zealand; 3 AgResearch Limited, Hamilton, 3240 New Zealand

**Keywords:** Ecology, Ecological genetics, Genetic variation

## Abstract

CRISPR gene drives have potential for widespread and cost-efficient pest control, but are highly controversial. We examined a potential gene drive targeting spermatogenesis to control the invasive common wasp (*Vespula vulgaris*) in New Zealand. *Vespula* wasps are haplodiploid. Their life cycle makes gene drive production challenging, as nests are initiated by single fertilized queens in spring followed by several cohorts of sterile female workers and the production of reproductives in autumn. We show that different spermatogenesis genes have different levels of variation between introduced and native ranges, enabling a potential ‘precision drive’ that could target the reduced genetic diversity and genotypes within the invaded range. In vitro testing showed guide-RNA target specificity and efficacy that was dependent on the gene target within *Vespula*, but no cross-reactivity in other Hymenoptera. Mathematical modelling incorporating the genetic and life history traits of *Vespula* wasps identified characteristics for a male sterility drive to achieve population control. There was a trade-off between drive infiltration and impact: a drive causing complete male sterility would not spread, while partial sterility could be effective in limiting population size if the homing rate is high. Our results indicate that gene drives may offer viable suppression for wasps and other haplodiploid pests.

## Introduction

CRISPR gene drives have been widely proposed as a promising potential technology for pest control or even eradication^1–3^. This technology provides an ability to disperse genetically engineered or altered genes throughout pest populations with much higher efficiency and prevalence than would be possible via normal genetic inheritance, even with genetic modifications that are deleterious for individuals and populations. CRISPR-Cas9 is an endo-nuclease system that will produce a targeted double-strand break in a DNA sequence based on complementarity to a guide RNA (gRNA) homing segment of ~ 20-bp^4^. A CRISPR-Cas9-generated double strand break can be repaired via homology-directed repair with a sequence with complementarity to the damaged region, converting heterozygous individuals for the mutation into homozygotes. This technology could be used to spread genetic variants through a population^5^. Some researchers highlight the possibility that gene drives could revolutionize pest control, making cost-effective eradication from islands or continents achievable^3,6,7^. Others recognise that issues such as genetic variation within a pest population might render gene drives ineffective^8,9^, or that gene drives could lead to unwanted global extinction of a species if the modified organism spreads widely^10^.

Understanding gene drive efficacy and the risks they pose is critical information required to provide an informed global debate and discussion. Detailed knowledge of target genes and their variation within native and introduced range populations are essential to predict resistance development and the usefulness or efficacy of gene drives. In vitro tests are needed to understand the efficacy of the CRISPR-Cas9 activity in genetically-variable populations, at non-target sites within the genome, as well as against related, non-target species. Simulation models are also required to predict the efficacy of the drive, as an understanding of the demography of the targeted population is crucial before the viability of gene-drive suppression can be critically and appropriately assessed^11^. Previous models for gene drives have assumed a diploid mating system, yet haplodiploid species comprise around 15% of the arthropods^12^. Many models focus on change in allele frequencies as a gene drive alleles spread^13,14^, whereas regulating population size or eradication is a key goal for pest management^11,15,16^.

**Figure 1:**
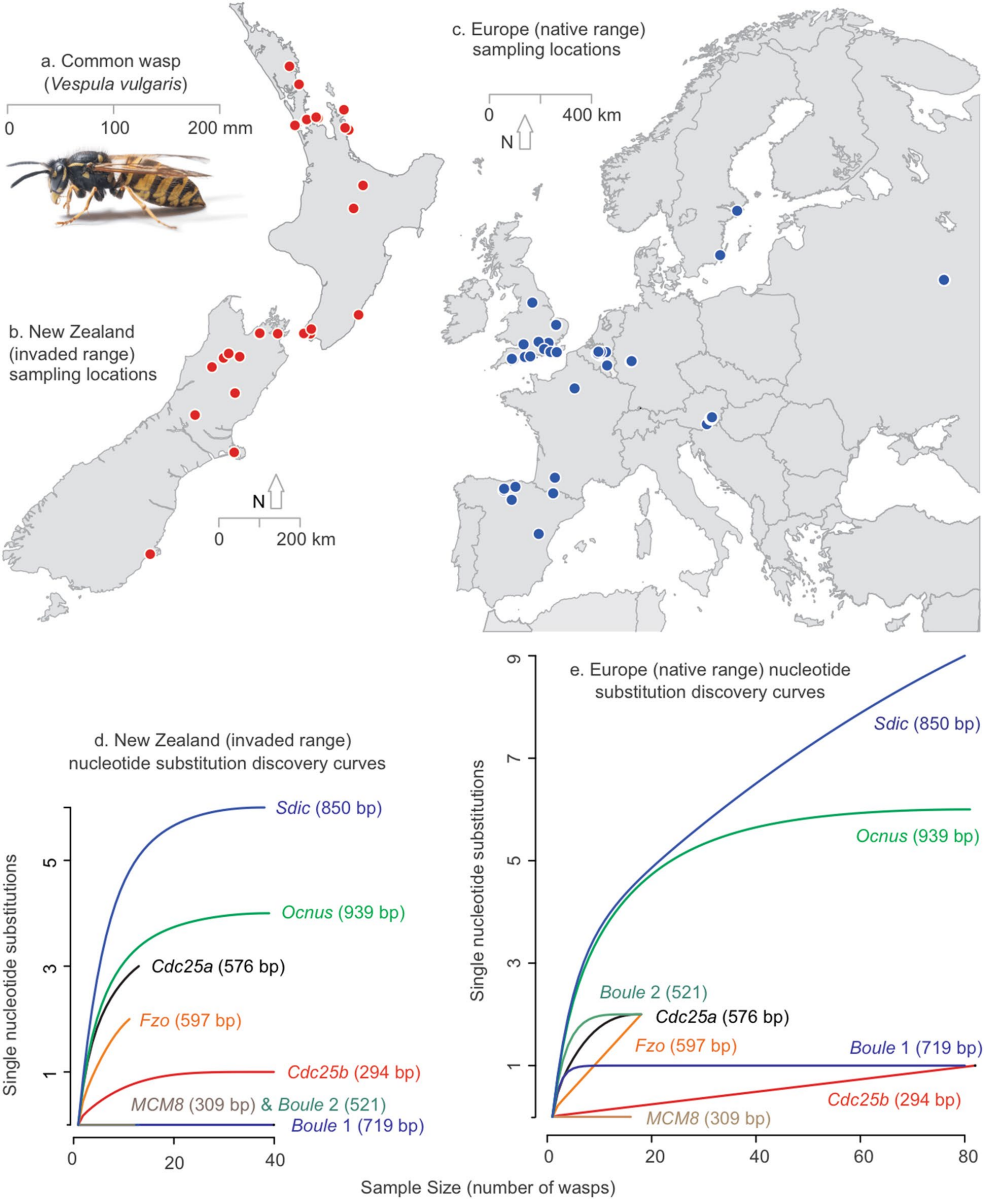
Sampling sites for the genetic analysis, and genetic variation of genes involved in spermatogenesis in each region. (**a**) The common wasp is native to Eurasia but is now widespread throughout New Zealand, especially in the upper South Island in native honeydew beech tree forest communities. Photograph by Colin McDiarmid. Sites where wasps were sampled from (**b**) the introduced range in New Zealand and (**c**) the native range. (**d**) and (**e**) show a rarefaction analysis of the discovery rate and diversity of nucleic acid substitutions within each spermatogenesis gene region from European and New Zealand samples. For some gene regions (e.g. *boule1*) there is little variation anywhere, while other genes show more substitutions in the native range than in New Zealand (e.g. *sdic*). *bp *base pairs in the gene sequence analysed.

Here, we provide a genetic and modelling analysis for a gene drive in the common wasp, *Vespula vulgaris* L (Fig. 1a). Common wasps have been included on the list of ‘100 of the World’s worst invasive alien species’^17^. Native to Eurasia, this insect has invaded Australia, South America, Hawaii and New Zealand^18^. Multiple invasions have occurred into the invaded ranges^19^. These wasps are generalist predators consuming 0.8–4.8 million loads of prey/ha in New Zealand^20^, compete with native species for resources^21,22^, and exert an annual cost of ~ NZ$133 million^18^. They occur at extremely high densities that occupy over more than a million hectares of native forest^18^, making the annual cost and environmental impact of using pesticides prohibitive. New approaches to their management are needed.

*Vespula* wasps are haplodiploid, as are many of the other insects listed in the list of 100 of the World’s worst invasive insect species, including the red imported fire ant (*Solenopsis invicta*) and sweet potato whitefly (*Bemisia tabaci*)^17^. In haplodiploid insects, haploid males are produced from unfertilized eggs while females develop from fertilized eggs and are diploid. The typical life cycle of *Vespula* spp. wasps involves queens mating with 2–3 males in autumn^23^ before overwintering alone. In spring, queens begin a nest by laying fertilized eggs that produce female workers. The queen must feed and tend these larvae until they become adult workers, after which the queen will not leave the nest again. The colony grows over the summer period with several cohorts of sterile workers until new queens and males are produced in autumn. Gene drives have been proposed for use in common wasp management, with spermatogenesis genes as targets^3,18^. Spermatogenesis genes could be altered in queen wasps to cause viable sperm production in males to fail, resulting in sterile male production. Several potential spermatogenesis genes with likely male-specific expression have been identified in wasps^24–27^. These targets include the *boule* gene, which has been found to be essential for the entry and progression of maturation divisions and sperm differentiation in haploid males^24^. Should a modified queen mate with wild-type males, fertilized worker eggs will be produced, culminating in queen and male production in autumn, all of which will carry the CRISPR cassette and thus propagate it to the next generation. Should a modified queen mate with a genetically-modified male, fertilization will fail and all offspring in spring will be male. This nest will fail and die in spring or early summer as males do not forage or aid in nest maintenance. Should a modified queen mate with a mixture of wild-type and genetically-modified males, a substantially smaller nest is likely to be produced resulting in genetically-modified reproductive offspring in autumn. Incomplete knock-down efficiency of spermatogenesis using the CRISPR-Cas9 system^28,29^ similarly might produce nests of reduced size. A spermatogenesis gene drive could thus potentially eradicate wasps or act in a “suppression drive” fashion^14^ to lower their abundance.

A gene drive based on spermatogenesis failure could have the key advantage that it would be self-propagating and cost-effective for large-scale control of invasive pests. An investigation of such an approach will also inform the use of gene drives for other haplodiploid species. A critical limitation on the use of gene drives in wasp and pest control is a ‘social license to operate’^3^, wherein government policy and the use of these technologies should have informed public support. There is concern regarding gene drives as a new and uncertain technology, particularly concerns over their specificity, and issues if genetically-modified wasps escape beyond New Zealand^4,30,31^. Our goal in this study was to provide data for these debates by assessing the potential for a gene drive to control wasp populations using spermatogenesis genes as targets, identifying potential risks with resistance. We were particularly interested in naturally occurring variation of spermatogenesis gene targets, as in-frame variation can induce drive resistance^32^. Alternatively, such intra-specific variation could also enable a precision drive^4^ that targets specific genotypes represented in genetically depauperate invasive populations, as found for the *Vespula* species in New Zealand^33,34^. Precision drives could offer a safeguard in that if genetically modified individuals return to their home range, few or a limited number of genotypes in the native range would be affected. We also examined intra- and inter-specific non-target effects and modelled predicted wasp population trends and control. Rather than focusing on allele frequencies^8,35,36^, our modelling was based on effects on wasp numerical abundance for suppression or eradication^32,37–39^. We believe our study will inform the global debate on the use of gene drives for pest control as well as providing some of the risk/benefit data required for assessment of these technologies.

## Results and discussion

### Variation in eight spermatogenesis gene regions

We examined populations of common wasps from their native range (n = 83) and New Zealand (n = 43; Fig. 1b,c; Supplementary Table 1) for variation in eight gene regions predicted to be associated with spermatogenesis (Supplementary Table 2). Targets were chosen on the basis of a literature search for genes in other haplodiploid species that have been found to be involved in sperm production. Six genes were examined, but for two genes two regions were examined (*boule* and *cdc*), which could be useful in situations when multiple sgRNAs are used to reduce the generation of in-frame resistance alleles^32^. All eight regions showed some degree of variation, with a differing numbers of single nucleotide polymorphisms (SNPs) (Fig. 1d,e). Most of the genetic variation occurred in the native range, consistent with previous observations of considerably higher levels of intra-specific genetic diversity of *Vespula* wasps in the native range compared to the introduced populations^19,33,34^. A substantial reduction in genetic variation in the introduced range, relative to the native, is expected for many invasive species and could facilitate the development of precision drives. A precision drive would allow the CRISPR-Cas9 system to target only certain genotypes within the native and invaded ranges^4^, providing some assurance that an entire species would not be affected if modified wasps were to be introduced back to their native range.

Our preliminary screening determined that some of the loci had no variability, some loci presented some variability, and only one locus had a number of substitutions. From the initial eight gene regions examined, we selected four genes to sequence for all wasp samples collected. *Boule protein, region 1* (*boule1*) and *cell division cycle 25, region B* (*cdc25B*) showed only a single SNP each, *ocnus* presented six substitutions, and *sperm-specific dynein intermediate chain* (*sdic*) presented nine SNPs and a 6-base pair indel in individuals from Austria, the UK and in many New Zealand and Russian wasps (Fig. 1). These results indicate that there is potential to select different target genes that demonstrate a substantial range of intra-specific variation. *Boule* being highly conserved and displaying little intra-specific variation implies that it has a strongly-selected, essential function. Targets with essential functions such as this would present a limited opportunity for the selection of wasp genotypes resistant to the guide-RNA. This limited variation also represents the highest probability for deleterious effects on the entire species should genetically-modified individuals be returned to their home range. Genes such as *sdic* show the opposite scenario. The intra-specific variation in this target offers an opportunity for a precision drive that could offer a degree of safeguard should genetically-modified individuals be returned to Europe: some genotypes within the home range would be affected, but not all. The variation in *sdic* might also mean there is unsampled variation present in the invaded range, leading to resistance, and potentially, eventual drive inefficacy.

The development of resistance alleles that could render the gene drive useless is of main concern. If a drive was to be deployed, our preference would be to use a ‘precision drive’ with genes such as *ocnus* or *sdic*. The use of multiple sgRNAs (single guide RNAs) or multiplexing to these targets could increase the effective homing rate and decrease the rate of resistant allele generation^11,37^. Other suggestions to limit resistance development include tightly regulated promoters to restrict nuclease expression to the early germline^9^. If resistance did arise, the solution could be as simple as re-designing the sgRNA to account for this variation. Rapid and efficient genetic transformation in insects is becoming possible through new approaches in CRISPR-Cas9 editing^30^. Exploiting the observed genetic variation between ranges into a daisy-chain drive system^31^ could offer substantial additional levels of safety.

### In vitro CRISPR-Cas9 testing and non-target effects

Three spermatogenesis gene sequences (*boule1*, *sdic* and *ocnus*) were chosen for use in in vitro CRISPR-Cas9 experiments. All sgRNA designs are presented in Supplementary Table 3. The sgRNA designed for *boule1* encompassed a DNA sequence that was invariant across all wasp samples in the native and invaded range. Two sgRNA versions for *sdic* targeted the region around the 6-base pair indel in individuals present in New Zealand and the native range: one version targeted samples with the observed indel, and the other targeted samples without the indel. Two sgRNA versions were tested for *ocnus*: one version targeted wasps with a SNP at position 266 found in countries from the native range including Russia and Spain, while the other version targeted wasps without the SNP that included all wasp genotypes from New Zealand. A BLASTn search optimised for small query sequences indicated that these sgRNA sequences had no homology elsewhere within the common wasp genome. Our in vitro test examined for CRISPR-Cas9 cleavage of PCR products amplified from the *boule1*, *ocnus* and *sdic* genes in 10 samples from the native range, and 10 from New Zealand.

The five CRISPR-Cas9 assays for the three spermatogenesis genes showed varying results that were dependent on the sgRNA design. The sgRNA targeting *boule1* cleaved this gene in all 20 *V. vulgaris* samples tested (Supplementary Table S7; Supplementary Fig. 1), which was expected due to the absence of any variation within this gene across samples. The two *sdic-*targeted sgRNA assays showed indel-dependent results. sgRNA assays that targeted samples without the 6-bp deletion effectively cleaved those samples, but also resulted in partial cleavage of the gene from wasp samples that were indel-positive (Supplementary Fig. 2). Assays using sgRNAs designed for indel-positive samples worked in a highly targeted fashion. This indel-positive sgRNA cleaved only indel-positive samples, although the cleavage was incomplete (Supplementary Fig. 3). The two *ocnus* sgRNA assays were designed to discriminate between differences in one SNP. The first assay used sgRNA targeting the Cas9 cleavage to samples with the SNP and showed specificity, where only SNP-positive samples where cleaved and remaining samples were unaffected (Supplementary Fig. 4). However, contrary to our expectations the second sgRNA design that targeted SNP-negative samples, did not show any specificity. This sgRNA cleaved the *ocnus* gene in all samples that we analysed (Supplementary Fig. 5). That the *ocnus* SNP gave better precision and efficacy than the *sdic* indel was unexpected, and we cannot explain why the SNP would be more effective than an indel. This emphasizes the importance of *in-vitro* testing of sgRNAs before development of transgenics.

Our results provide confidence that a precision drive could be implemented for common wasp control. Additional and more extensive sampling of genes within native and invasive populations could identify SNPs or indels allowing efficient specificity for invaded populations, with a higher proportion of the native populations being unaffected. Quantification of the sgRNA assays showed considerable variation with the highest cleavage levels (93.78 ± 0.87%; mean ± SE) in the *ocnus* SNP-targeting assay and the *sdic* indel positive-targeting assay with the lowest rate of 46.59 ± 22.17% (Supplementary Fig. 3).

One of three key issues in proposing gene drive targets is understanding target specificity^10,14,40,41^. In the unlikely event of hybridization, would there be any possibility for the CRISPR-Cas9 modified wasps to affect other species? For our specificity analysis, we first bioinformatically compared the five sgRNA sequences for the *boule1*, *ocnus* and *sdic* spermatogenesis genes for the common wasp, with those from the genomes in the related species *V. germanica, V. pensylvanica*, the paper wasp *Polistes dominula*, and two bee species *Apis mellifera* and *Bombus terrestris*. A BLASTn search specific for each of our five 23-base pair sgRNA designs showed variable homology, depending on the gene target and species. None of the five gRNA sequences were homologous with any sequence on the *A. mellifera, B. terrestris* or *P. dominula* genomes. The two sgRNA sequences designed for *boule1*, and one for *sdic*, had identical targets on chromosome 4 of all three *Vespula* species. Similarly, the sgRNA targeting *ocnus* was conserved and present on chromosome 12 of all *Vespula.* The only sgRNA showing a high degree of specificity was the sgRNA targeting the indel of *sdic*, which did not correspond to sequences on any genome examined (the *V. vulgaris* genome sequenced was from a nest in New Zealand without this indel). This genomic analysis supported a recent phylogenetic analysis for these species^42^, with spermatogenesis genes for common wasp demonstrating a high degree of similarity to *V. germanica* while being substantially different to the bees *A. mellifera* and *B. terrestris* (Supplementary Fig. 6)*.*

Finally, where available we amplified the spermatogenesis gene regions *boule1, ocnus*, and *sdic* genes for all five non-target species for in vitro tests using our sgRNA in a CRISPR-Cas9 experiment (no equivalent genes were observed in some of the species). Our in vitro laboratory experiments supported the genomic bioinformatic analysis. The assays using the five designed sgRNA showed no indication of any gene cleavage for any non-target species outside of the *Vespula* genus (Supplementary Fig. 7). Gene cleavage was observed within all three *Vespula* species examined for sgRNA designed for *boule1*, one of the two *sdic*, and one of the two *ocnus* genes (Table 1). The other *sdic* sgRNA was specific to the common wasp samples. The remaining *ocnus* sgRNA designs were specific to genotypes within common wasps. Clearly, the specificity of a gene drive for pest management will depend highly on the gene region targeted and the sgRNA design. It appears possible to design multiple sgRNAs that can be specific to genotypes within a species, or that could function across different species within a genus.

**Table 1:** A summary of the sgRNA and CRISPR-Cas9 assay on the target species (common wasps or *Vespula vulgaris*) and four non-target hymenopteran insects. Equivalent genes were found in all species for *sdic v1* and *sdic v2*, but not for all of the other genes in all species. No gene was cleaved by the sgRNA in species outside of the social wasp genus *Vespula*. Within the *Vespula*, some sgRNA cleaved gene regions in all species, while other sgRNA was genotype-specific with common wasps. Gels are presented below that show results specific to each assay. ✕ = no equivalent gene found, or no cleavage observed; ✓ = homologous gene observed, or gene cleavage; * ✓= genotype specific cleavage.

Insect		Gene region and sgRNA version				
		*boule v1*	*ocnus v1*	*ocnus v2*	*sdic v1*	*sdic v2*
*Vespula vulgaris*	Equivalent gene found?	✓	✓	✓	✓	✓
(Common wasp)	Gene cleaved using sgRNA?	✓	✓*	✓	✓	✓*
*Vespula germanica*	Equivalent gene found?	✓	✓	✓	✓	✓
(German wasp)	Gene cleaved using sgRNA?	✓	✕	✓	✕	✓
*Vespula pensylvanica*	Equivalent gene found?	✓	✓	✓	✓	✓
(Western yellowjacket)	Gene cleaved using sgRNA?	✓	✕	✓	✕	✓
*Polistes dominula*	Equivalent gene found?	✓	✕	✕	✓	✓
(European paper wasp)	Gene cleaved using sgRNA?	✕	✕	✕	✕	✕
*Bombus terrestris*	Equivalent gene found?	✓	✕	✕	✓	✓
(Buff-tailed bumble bee)	Gene cleaved using sgRNA?	✕	✕	✕	✕	✕
*Apis mellifera*	Equivalent gene found	✕	✕	✕	✓	✓
(Western honey bee)	Gene cleaved using sgRNA	✕	✕	✕	✕	✕

### Population models

We developed population models for gene drive in common wasps based on spermatogenesis knockdown. Diploid wasp queens are univoltine. Queens mate two or three times^23^ with haploid males before overwintering alone. In some vespids fecundity and nest size depend on the number of viable sperm collected^43^, so we investigated a range of feasible alternatives in the context of full and partial drone sterility. These different approaches suggest that drives could function in an “eradication drive” or a “suppression drive” fashion, which are designed to extirpate or decrease the size of a population, respectively^14^. The models are based on the introduction of 100 wasp queens at time 0 into a 1 km^2^ area of New Zealand forests containing an average of 13.5 nests ha^−1^^44^. Integer-based versions of models, which included stochasticity and gene drift, produced similar results to our deterministic models (Supplementary modelling methods and results).

**Figure 2:**
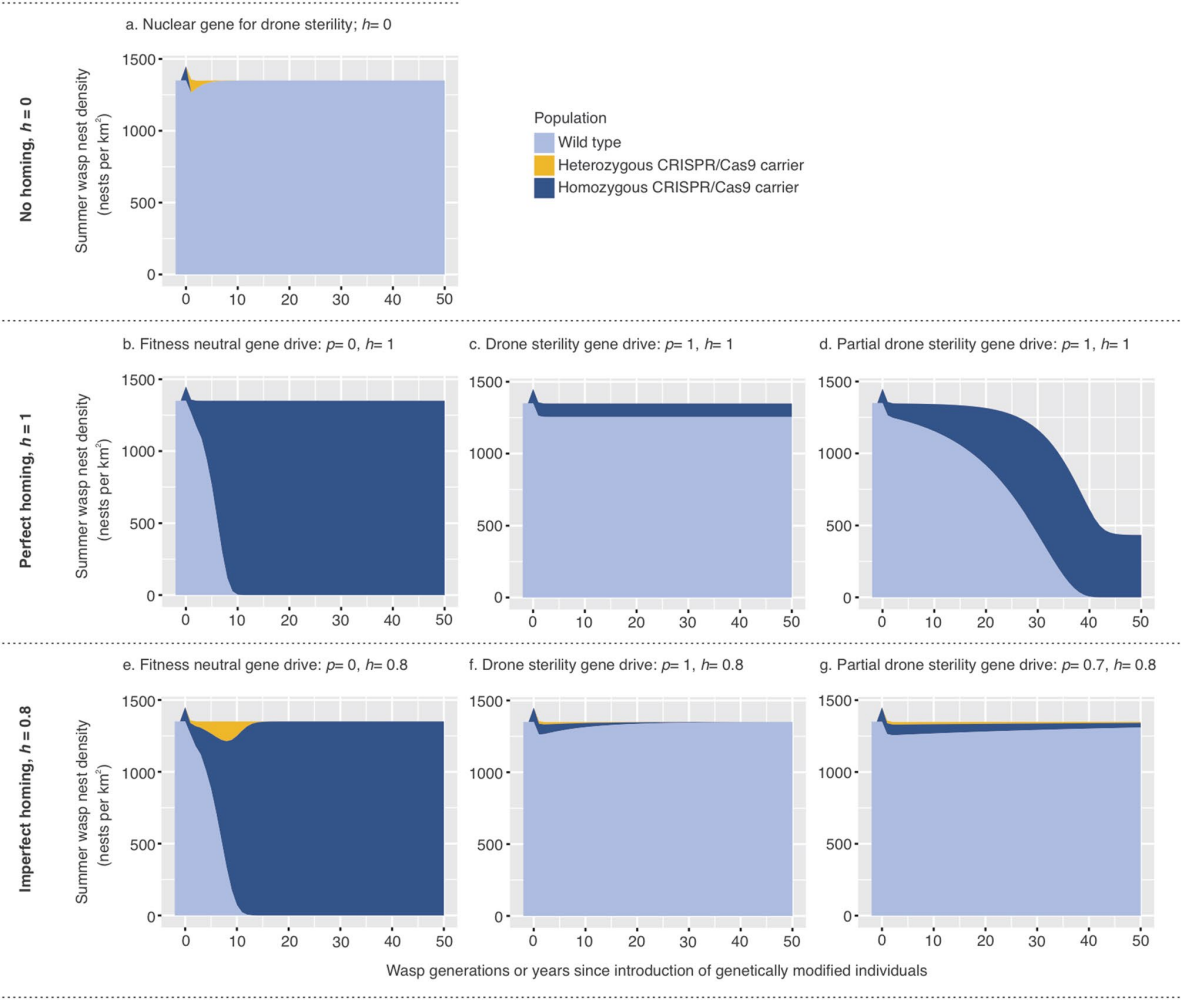
Model projections from a one-off introduction of 100 carrier queens/km^2^ into wasp populations. Light blue denotes wild-type queens, gold are heterozygous carriers, and dark blue indicates homozygous gene drive carriers. (**a**) A genetic modification without the CRISPR-Cas9 cassette is quickly lost from the population. (**b**) A CRISPR-Cas9 modification of a gene that would not influence wasp fitness sweeps through the entire population after 10 generations or years. A genetic modification causing complete sterility, in a gene drive scenario (**c**), persists in the population but doesn’t influence overall wasp densities. Partial drone sterility cases (**d**) assumes only 70% of matings with carrier drones are successful. Imperfect (80%) homing has little effect on a fitness-neutral drive (**e**), but may prevent a sterility drive from spreading sufficiently to impact the population (**f**,**g**).

As expected, the models indicated that a nuclear gene for drone sterility without a CRISPR cassette would be rapidly eliminated (Fig. 2a), but a fitness-neutral gene introduced with a CRISPR cassette could take as little as a decade (ten queen generations) to almost fully infiltrate the population (Fig. 2b,e). These results are similar to the predicted dynamics of Y-chromosome-linked modifications in male heterogametic species, that are designed to disrupt the fitness of female descendants^45^. Infiltration took longer with lower values for the homing rate and/or mating competitiveness of carrier drones. The potential for rapid spread of fitness neutral drives is consistent with other models (33). However, the models showed that gene drives causing complete male sterility would not spread (Fig. 2c), as the gene drive would be opposed by its loss through the sterility of male carriers. With perfect homing this balance results in a stable allele frequency: since male carriers are sterile offspring arise only from matings with non-carrier males, and the homing effect ensures all homozygous female carriers produce carrier offspring, while heterozygous carriers produce half homozygous carriers and half non-carriers. Therefore, the allele frequency does not change from one generation to the next except through drift or human management of wasp populations. If the homing rate is less than perfect, however, there will be a tendency for the proportion of carriers in the population to decline each generation, and the CRISPR cassette will eventually be eliminated (Fig. 2f).

**Figure 3:**
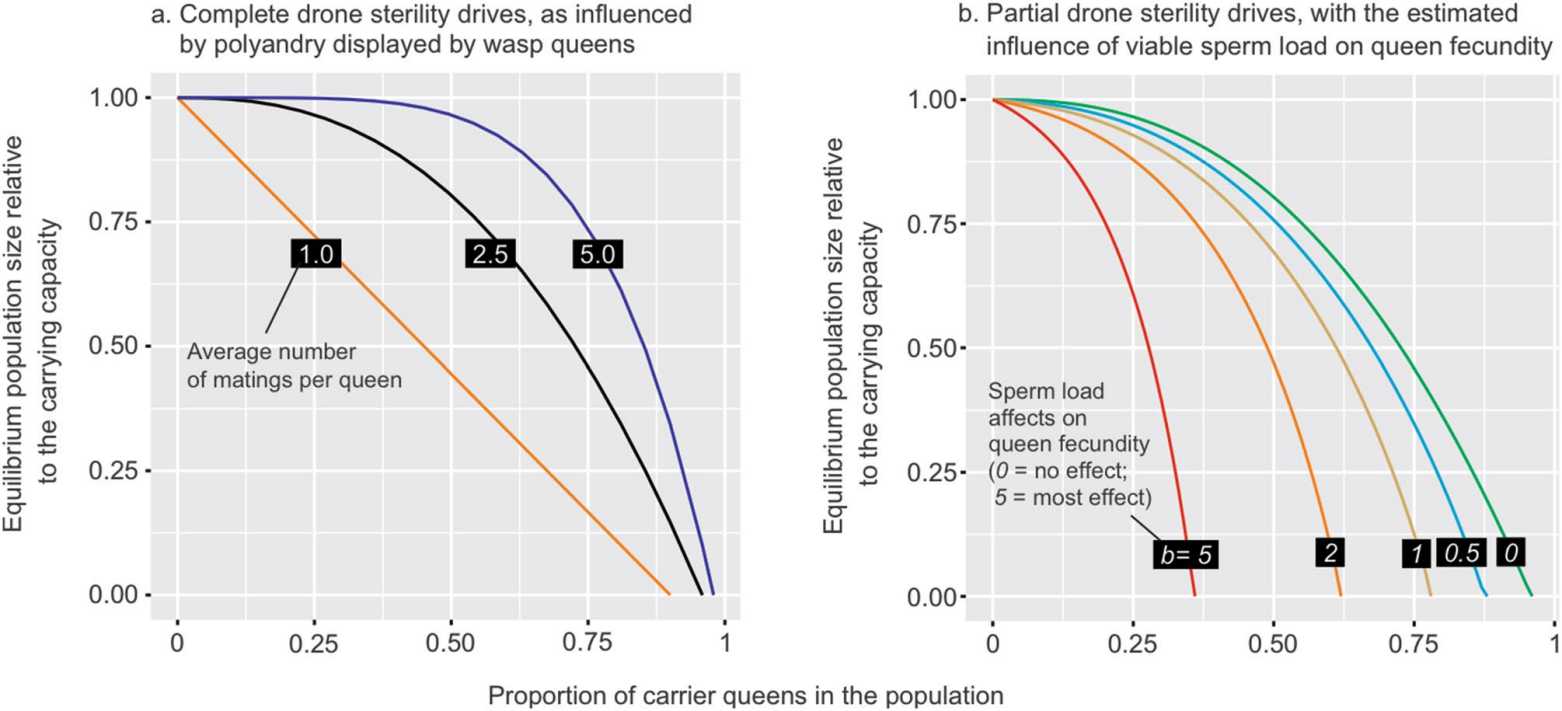
The effect on population size of the proportion of queens in the population that carry a genetic modification for complete or partial drone sterility. (**a**) With a high degree of polyandry displayed by females, a larger proportion of females in the population will need to be carriers of genetic modification before a substantial reduction in population size is achieved. We estimate that queens mate, on average, with 2.5 males in New Zealand^23^. (**b**) The volume of viable sperm collected by queens during mating may affect nest size and hence fecundity in *Vespula* wasps^43^. Increasing effects of viable sperm load on queen fecundity mean that a smaller proportion of the carrier queens is needed to influence wasp population size.

**Figure 4:**
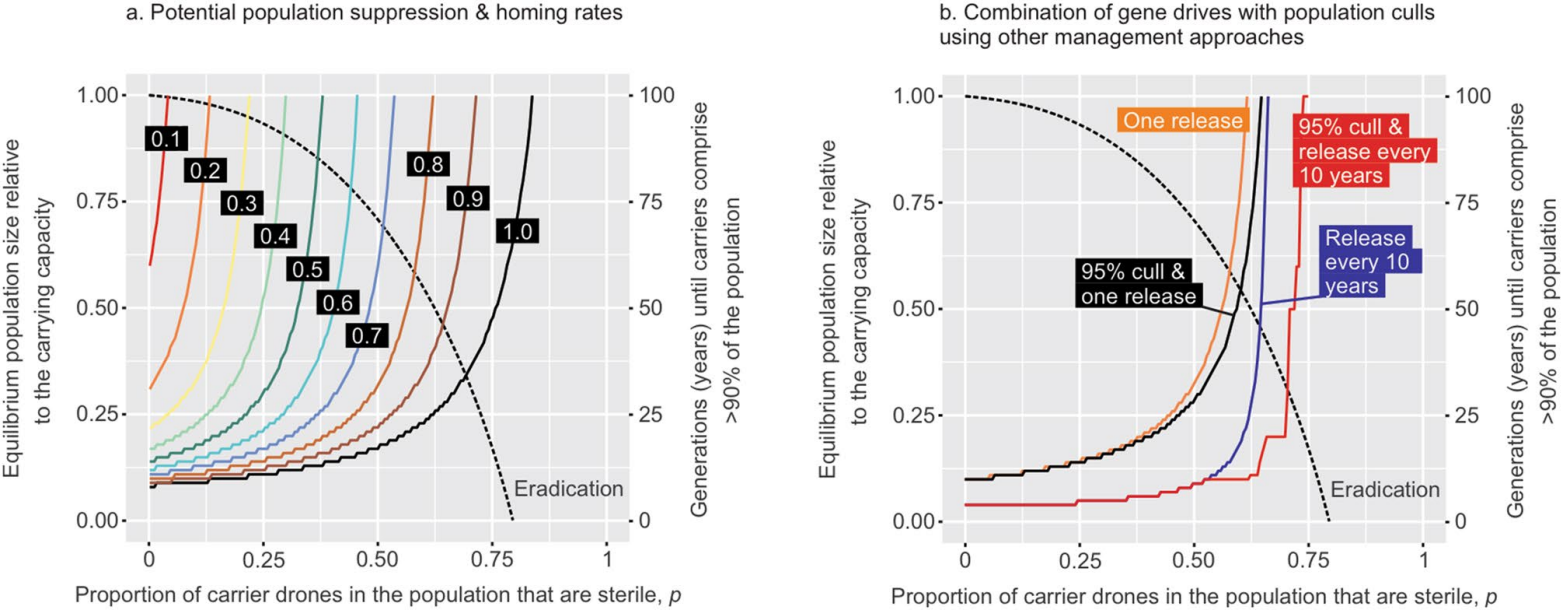
Effects of a gene drive for partial drone sterility. (**a**) The potential population suppression (dotted line, left axis) caused by a gene drive for partial drone sterility is not affected by the homing rate, but the time taken for the drive to spread through > 90% of the population (solid lines, right axis) is. Thus, eradication is theoretically possible when *p* > 0.8, but only if the homing rate is sufficiently high to allow the drive to spread (*h* > 0.92). Even with perfect homing (*h* = 1) spread and subsequent eradication is predicted to require about a century. (**b**) Gene spread can by facilitated by other management interventions. Here a drive with *h* = 80% homing is released once (orange line), once immediately following a 95% cull (black line), every 10 years (blue line), or every ten years after a 95% cull (red line).

Of particular interest is the result that gene drives causing partial drone sterility (Fig. 2d) may be more effective than those for complete sterility (Fig. 2c), because they allow the drive to spread naturally and subsequently reduce the population, providing the homing rate is sufficiently high (Fig. 2g). Allowing for some male carriers to breed breaks the deadlock between homing and drive loss through infertility. The amount by which the population is eventually reduced by partial gene sterility depends on the number of males each queen mates with (or their degree of polyandry; Fig. 3a) and the degree to which relative sperm load affects queen fecundity (Fig. 3b). However, in the partial sterility simulations, like the full-sterility model, the greater the population suppression, the longer it takes for the gene to spread. Such an effect with gene drive spread and effects has previously been likened to relatively non-virulent pathogens spreading, while more virulent pathogens with high fitness effects fail to spread or suppress populations^39^. Incorporating realistic population demography into simulation models, such as polygynous mating and partial drone sterility (Fig. 4), can substantially alter predictions of drive success for pest eradication and suppression outcomes^11,46^.

**Figure 5:**
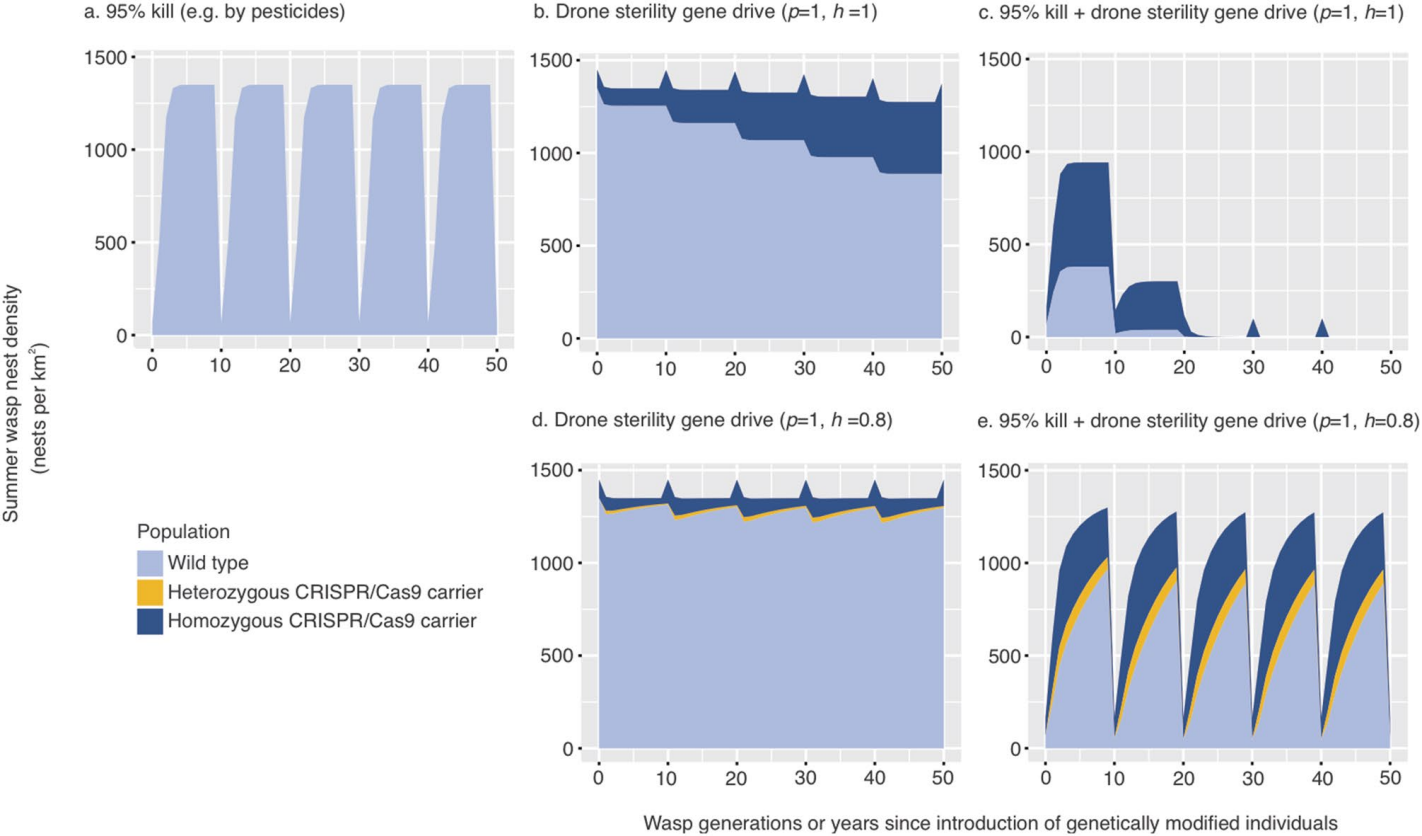
The impacts of ten-yearly wasp management interventions on modelled wasp population. (**a**) Simulations showing how management solely by toxic baits or poisoning causing 95% kill of queens and nests, though populations quickly recover. (**b**) Repeated introductions of 100 queens carrying the CRSIPR-Cas9 modification for complete drone sterility cause an increased abundance of genetically modified individuals but has little influence on overall abundance. (**c**) A scenario where control by toxic baiting and genetically modified wasps are introduced every 10 years. (**d**) and (**e**) comparatively show how suppression is dependent on the homing rate being sufficiently high.

Despite the tendency not to spread, a drone sterility drive may still have an impact on population size if it can be made sufficiently abundant in the population. One potential way to achieve this in practice is to release carrier queens into a population that has been temporarily reduced. Neither 95% effective poisoning at 10-yearly intervals (Fig. 5a) nor regular release of gene drive carriers (Fig. 5b) would make a long-term impact on wasp population size, but a combination of both could cause extinction if the homing rate is sufficiently high (Fig. 5c). The key is to reach the critical threshold for carrier abundance whereby the sterilizing effect of the gene drive exceeds the population’s reproductive potential. Analytical estimates for this threshold (Supplementary modelling methods and results) depend in part on the degree of polyandry (Fig. 3). For wasps, the default parameter values suggested that eradication would require at least 96% of queens to carry the drone sterility allele (Fig. 5a–e). Other modelling approaches have recently also found that in principle, CRISPR driver alleles can spread in pest populations of haplodiploid species across a wide range of conditions^47^. In drives with a high fitness cost, as suggested in our modelling work, a high conversion rate would be needed to successfully fix the modified germline allele. The conditions favouring the spread of genetically modified alleles have been suggested to likely be narrower in haplodiploid than diploid species^47^ .

## Conclusions

Webber et al.^10^ identified three key issues in their discussion on whether CRISPR-based gene drives represent a “biocontrol silver bullet or global conservation threat”: (i) the importance of understanding target specificity, (ii) the implications of population connectivity, and (iii) the need to carefully consider unintended cascades for community dynamics. These concerns are echoed elsewhere^15,16,41,48,49^.

Regarding specificity, our analysis on spermatogenesis genes highlighted a range of potential targets. Some of these gene targets, such as *ocnus*, could enable drive intra-specific specificity and selection. Individual genotypes within a species and invaded range could be culled, but a careful design using the *ocnus* gene would provide a safeguard against an entire species being affected. There are no off-target sites in the common wasp genome that would be affected by a CRISPR-Cas9 cassette that used sgRNA based on our *ocnus* gene. The potential for de novo resistance development or mutation represents a risk in the use of gene drives, though methods of resistance management have been suggested^8,9,11,14^. Multiplexing additional population-specific sgRNA within the engineered cassettes for each target gene may overcome resistance issues^50^, with our analysis discovering multiple targets on the *ocnus* and *sdic* genes that provide such prospective sgRNAs. After gene drive release, population monitoring and potential redesign of sgRNAs could also account for this issue. A common question from public audiences is “what happens if a genetically modified wasp mates with a related wasp, or even a honey bee?”^40,41^. We have demonstrated for such an improbable hybridization event, utilizing intra-specific genetic variation would mean that even closely-related species are unlikely to be affected. Some conserved gene targets, such as *boule* represent more of a risk for non-target effects, though any potential non-target effects appear *Vespula* genus specific.

Connectivity between the invaded and native range will always be an issue with the use of gene drives. The safeguard of using intra-specific variation^4^ targeting only some genotypes could limit the native range risk. Techniques such as daisy drives could offer additional safeguards^31^. There have been at least six common wasp introduction events into New Zealand^19^, though it is unknown how many return events back to Europe have occurred. New Zealand as a remote island nation in a separate hemisphere provides a further safeguard. Nevertheless, the globe is highly interconnected by trade and the risk of a return of wasps is non-negligible. Common wasps are not universally hated in Europe and may play an important role in ecosystem function there^51,52^. Ideally, a fast extinction process resulting from a gene drive would reduce the potential and time for wasps to enter a trade pathway back to Europe. Our modelling analysis indicates, however, that some gene drive targets will not result in extinction or at least take several decades to achieve. The probability of genetically-modified wasps entering a trade pathway and returning to the native range increases with increasing time. We thus consider that population-specific gene targets in precision drives are more important in situations where pest suppression is likely, compared to eradication scenarios (when there is less of chance for genetically modified individuals of returning to their native range).

The most likely unintended cascade effect would be for invasive German wasps to increase in abundance, should common wasps be culled or their populations substantially reduced. Common wasps displaced German wasps from beech forests in New Zealand^53^, where they would almost certainly return. This scenario suggests simultaneous control of both *Vespula* species would be necessary. Other unintended cascades might include an increase in abundance in pest species such as flies or some forest defoliating insects. Unintended consequences of wasp removal are valid concerns, but we also note that New Zealand ecosystems evolved without any social wasp species.

Pest control or eradication is essential to limit biodiversity declines and extinctions, to which these wasps are contributing in New Zealand^54,55^. Any control method has potential problems and pitfalls and should be approached with care. Gene drives are a potential next-generation technology for pest control, including for wasps. Our modelling analysis indicated that a gene drive using spermatogenesis genes could result in population suppression, but eradication seems likely to require a combined approach with other control methods. This outcome highlights the need to carefully assess and exploit variation in target genes to limit the potential of genetically-modified wasps affecting populations in the native range. Such genetic variation clearly occurs. Further genetic analysis on different gene targets could identify targets with even higher specificity, which could also influence suppression or extinction goals differently if used in precision or daisy-drive^31^ fashion. Finally, we note that should a gene drive for these wasps be desired, significant challenges still lie ahead in the production of a transgenic wasp line. The efficient production of CRISPR-Cas9 modified hymenopteran insects including honey bees (*Apis mellifera*)^56^ and ants^57^ offers avenues for this work.

## Materials and methods

### Insect collection, DNA extraction, and spermatogenesis gene analysis

We collected *Vespula vulgaris* wasps throughout its native range in Eurasia (n = 83) and its introduced range in New Zealand (n = 43) to screen genetic variation in genes involved in the process of sperm production or sperm maturation. Samples were either collected fresh for this study, or were from a previous project examining genetic diversity using mitochondrial genes^19^. Specimen collection information is presented in Supplemental Table 1 and includes detail on the other hymenopteran species used in this project. Individuals were collected and immediately placed in 99% ethanol or frozen until DNA extraction. We extracted genomic DNA from wasps using a CTAB and chloroform based protocol. Briefly, we homogenised the whole wasp in a microcentrifuge tube with 1 mL of GENEzol plant DNA reagent (Geneaid Biotech, Taiwan) and 5 μL of β-mercaptoethanol (Sigma Aldrich, Michigan, USA) in a Precellys Evolution homogeniser (Bertin Instruments, France).

We initially screened approximately 30 individuals for eight gene regions of interest: *boule protein region 1*, *boule protein region 2*, *cdc25 region A*, *cdc25 region B*, *fuzzy onions*, *helicase MCM8-like, ocnus*, and *sdic* (Supplementary Table 2). Primers for these genes in *V. vulgaris* (Supplementary Table 4) were designed by aligning sequences for these genes available for other insect species on GenBank to a *V. vulgaris* draft genome. Each gene was amplified in 15 μL reactions containing 1 μL of template DNA, 0.5 μM forward primer, 0.5 μM reverse primer, 0.5 μL Bovine Serum Albumin (Sigma Aldrich, New Zealand), and 1 × MyTaq Mix (Bioline, London, UK). Each PCR product was examined by agarose gel electrophoresis and purified with rSap combined with Exo 1 (New England Biolabs, Ipswich, MA, USA). Sequencing was performed on an ABI 3130 × 1 Genetic Analyzer (Applied Biosystems, Foster City, CA, USA) at Macrogen Inc. (South Korea). We aligned gene sequences using the default alignment algorithm implemented in the software Geneious v. 10.2.6 (https://www.geneious.com). GenBank accession numbers for these sequences are MN088861–MN089473. See Supplementary Table 5 for detailed accession number information relating loci with specimens. The R package vegan^58^ was then used in a rarefaction analysis to infer the discovery rate and diversity of nucleic acid substitutions within each spermatogenesis gene region.

### CRISPR-Cas9 in vitro DNA cleavage assay

We designed sgRNAs for each gene (Supplementary Table 3). Synthetic sgRNAs (Invitrogen TrueGuide sgRNA, ThermoFisher Scientific) were diluted to 100 μM and stored at − 20 °C. Prior to the in vitro CRISPR-Cas9 assay, PCR products for 20 *Vespula vulgaris* samples were generated from *boule*, *sdic* and *ocnus* genes using a high-fidelity PCR system (Platinum SuperFi PCR Master Mix, ThermoFisher Scientific). In addition, PCR products were generated from non-target species *Vespula germanica* (*boule*, *sdic* and *ocnus*), *Vespula pensylvanica* (*boule*, *sdic* and *ocnus*), *Polistes dominula* (*boule* and *sdic*), *Bombus terristris* (*boule* and *sdic*) and *Apis mellifera* (*sdic*). Each 25 μL reaction contained 50 ng of template DNA, forward and reverse primers (Supplementary Tables 4 and 6) at final concentrations of 0.5 μM, and Platinum SuperFi PCR Master Mix. Reactions proceeded as follows: 98 °C for 30 s; 35 cycles of 98 °C for 10 s, 64.7 °C (*V. vulgaris boule*), 65 °C (*V. vulgaris sdic*) or 62.4 °C (*V. vulgaris ocnus*) for 10 s, 72 °C for 30 s; 72 °C for 5 min; 4 °C (hold). PCR products were purified using DNA Clean and Concentrator-5 columns (Zymo Research, CA, USA) and DNA concentrations were measured using a NanoPhotometer (Implen, Germany). PCR products were diluted to 30 nM with water. The CRISPR-Cas9 in vitro assay followed the manufacturers protocol described for Cas9 nuclease (New England Biolabs, MA, USA). In brief, reactions were assembled containing NEBuffer 3.1 (New England Biolabs, MA, USA), 30 nM sgRNA, 30 nM Cas9 and water to a final volume of 27 μL followed by preincubation for 10 min at 25 °C. Three μL of 30 nM PCR product was then added (final concentration 3 nM) and the reaction incubated at 37 °C for 15 min. The reaction was ended by adding Proteinase K (New England Biolabs, MA, USA) and incubating at room temperature for 10 min. To purify and concentrate the digested DNA, samples were loaded onto DNA Clean and Concentrator-5 columns (Zymo Research, CA, USA) and eluted in 10 μL of water. The samples were then resolved by 2% agarose gel electrophoresis for fragment analysis. The origin and details of the 10 wasps from the native range, and 10 from the invaded, are shown in Table S7 of the Supplementary Material.

### Population modelling for wasp population control

We derived and analysed a population model for a gene drive affecting spermatogenesis in *Vespula* wasps, as described in full in the Supplementary modelling methods and results. Summer queen density, equivalent to nest density, is denoted *Q*, with subscripts specifying the genotype of the queens and of the drones with which they mated the previous autumn (mating only occurs in autumn and, if any queens fail to successfully mate—the frequency of which is unknown—their nests will fail in the following spring). Hence the three diploid queen genotypes (*ww*, *wi* and *ii*, where *w* denotes the WT gene and *i* the modified gene) and two haploid drone genotypes (*w* and *i*) lead to six possible queen types in summer: *Q*_*ww_w*_, *Q*_*ww_i*_, *Q*_*wi_w*_, *Q*_*wi_i*_, *Q*_*ii_w*_, and *Q*_*ii_i*_. The drones *D* produced in the subsequent autumn,

$$D_{w} \propto Q_{ww\_w} + Q_{ww\_i} + \frac{1}{2}\left( {Q_{wi\_w} + Q_{wi\_i} } \right)$$

$$D_{i} \propto \frac{1}{2}\left( {Q_{wi\_w} + Q_{wi\_i} } \right) + Q_{ii\_w} + Q_{ii\_i}$$

mix widely and mate with the autumn gynes (new queens) at random. Assuming the spermatogenesis gene drive results in only a proportion *p* of matings with carrier drones transferring viable sperm, the proportion of gynes being successfully fertilised is closely approximated as

$$f = 1 - \left( {\frac{{pcD_{i} }}{{D_{w} + cD_{i} }}} \right)^{m}$$

where *m* = 2.5 is the average number of matings per gyne^23^ and *c* is the relative competitiveness of carrier drones. Furthermore, the proportion of fertile matings that lead to the WT allele being passed on to offspring is

$$j = \frac{{D_{w} }}{{D_{w} + c(1 - p)D_{i} }}$$

Old queens die in winter, but a proportion *s* = 0.02 of the gynes survive to compete for nest sites in spring. These processes are combined in a single density-dependent survival factor

$$g = \frac{s}{{1 + \frac{s\Sigma G}{n}}}$$

where *n* = 1,500 km^−2^ is a nest site competition factor resulting in a summer nest density of 1,350 km^−2^ (40). Importantly, unmated queens participate in nest site competition but are unable to produce diploid workers, so these nests subsequently fail. Here, Σ*G* is the total density of gynes that were produced in autumn and is given by *λZ*^*b*^(*Q*_*ww_w*_ +* Q*_*wi_w*_ + *Q*_*ii_w*_ + (1 −* p*)(*Q*_*ww_i*_ + *Q*_*wi_i*_ + *Q*_*ii_i*_)) where the potential number of gynes produced per nest *λ* = 560 is modified by a power function of the sperm load carried by queens (38)

$$Z \cdot = \frac{{D_{w} + c(1 - p)D_{i} }}{{D_{w} + cD_{i} }}$$

To avoid the use of additional subscripts, we use a dot (·) to indicate values in the following generation *t* + 1. Hence sperm load potentially affects the number of progeny produced in the following generation. Since there is exactly one nest per queen, the model for nest density from one summer to the next (indicated by a dot) is:

$$Q_{ww\_w} \cdot = \lambda Z^{b} fgj\left( {Q_{ww\_w} + \frac{1}{2}Q_{wi\_w} } \right)$$

$$Q_{wi\_w} \cdot = \lambda Z^{b} fgj(1 - h)\left( {(1 - p)\left( {Q_{ww\_i} + \frac{1}{2}Q_{wi\_i} } \right) + \frac{1}{2}Q_{wi\_w} + Q_{ii\_w} } \right)$$

$$Q_{ii\_w} \cdot = \lambda Z^{b} fgj\left( {(1 - p)\left( {hQ_{ww\_i} + \frac{(1 + h)}{2}Q_{wi\_i} + Q_{ii\_i} } \right) + h\left( {\frac{1}{2}Q_{wi\_w} + Q_{ii\_w} } \right)} \right)$$

$$Q_{ww\_i} \cdot = \frac{(1 - j)}{j}Q_{ww\_w} \cdot$$

$$Q_{wi\_i} \cdot = \frac{(1 - j)}{j}Q_{wi\_w} \cdot$$

$$Q_{ii\_i} \cdot = \frac{(1 - j)}{j}Q_{ii\_w} \cdot \cdot$$

We explored this model for: a normal gene (*h* = 0) causing complete (*p* = 1) or partial (0 < *p* < 1) drone sterility; a gene drive (*h* > 0) potentially affecting drone mating competitiveness (*c* ≤ 1) but with no effect on drone fertility (*p* = 0); a gene drive (*h* > 0) causing complete drone sterility (*p* = 1); and a gene drive (*h* > 0) causing partial drone sterility (*p* < 1). We also simulated integer-based versions of the models to include the effects of gene drift. Full details are given in the Supplementary Material.

## Supplementary information

Supplementary Information 1.

## Data Availability

Additional information including additional methods and materials, results and Genbank accession numbers can be found in the file Supplementary Information.
