# Supplementary material for: The potential for a CRISPR gene drive to eradicate or suppress globally invasive social wasps
Source: Sci Rep. 2020 Jul 24;10:12398. doi: 10.1038/s41598-020-69259-6 (PMC7382497; doi:10.1038/s41598-020-69259-6)
Supplement: Supplementary file 1 — Supplementary Information 1. [file 41598_2020_69259_MOESM1_ESM.docx]

Supplementary Information for

**The potential for a CRISPR gene drive to eradicate or suppress globally invasive social wasps**

**Philip J. Lester^1*^, Mariana Bulgarella^1^, James W. Baty^1^, Peter K. Dearden^2^, Joseph Guhlin^2^, John M. Kean^3^**

**1**  School of Biological Sciences, Victoria University of Wellington, PO Box 600, Wellington, New Zealand. **2** Genomics Aotearoa and Biochemistry Department, University of Otago, Dunedin, New Zealand. **3** AgResearch Limited, Hamilton 3240, New Zealand.

*To whom correspondence may be addressed. Email: Phil.Lester@vuw.ac.nz

**This PDF file includes:**

Tables S1 to S7

Figures S1 to S7

Supplementary text for the modelling methods and results

Figures S8 to S23

Table S8

Glossary

SI References

**Table S1.** Locality and specimen information for the *Vespula vulgaris* individuals and for the five non-target hymenopteran species included in this study.

| **Species** | **Sample ID** | **Locality** | **Country** | **Date** | **Latitude** | **Longitude** |
| --- | --- | --- | --- | --- | --- | --- |
| *Vespula vulgaris* | AU125 | Gschwendt, Kumberg | Austria | 9-Oct-18 | 47.178 | 15.573 |
| *Vespula vulgaris* | AU126 | Purgstall bei Eggersdorf | Austria | 14-Oct-18 | 47.124 | 15.584 |
| *Vespula vulgaris* | AU127 | Tegetthofplatz, Graz | Austria | 19-Oct-18 | 47.077 | 15.459 |
| *Vespula vulgaris* | AU131 | Brendlhütte, Oberfresen | Austria | 1-Nov-18 | 46.767 | 15.074 |
| *Vespula vulgaris* | AU132 | Kainbach bei Graz | Austria | 4-Nov-18 | 47.096 | 15.525 |
| *Vespula vulgaris* | AU135 | Graz | Austria | 8-Nov-18 | 47.090 | 15.437 |
| *Vespula vulgaris* | BEL1L5 | Leuven | Belgium | 2015 | 50.829 | 4.869 |
| *Vespula vulgaris* | BEL2L5 | Leuven | Belgium | 2015 | 50.805 | 4.787 |
| *Vespula vulgaris* | BEL2H5 | Zonhoven | Belgium | 2015 | 50.993 | 5.372 |
| *Vespula vulgaris* | BEL3 | Heverlee | Belgium | 18-Sep-12 | 50.843 | 4.671 |
| *Vespula vulgaris* | BEL17B6 | Leuven | Belgium | 2016 | 50.889 | 4.639 |
| *Vespula vulgaris* | BEL19B6 | Leuven | Belgium | 2016 | 50.889 | 4.639 |
| *Vespula vulgaris* | BEL21B6 | N Brussels airport | Belgium | 2016 | 50.914 | 4.479 |
| *Vespula vulgaris* | BEL22B6 | Leuven | Belgium | 2016 | 50.821 | 4.888 |
| *Vespula vulgaris* | BEL23B6 | Aarschot | Belgium | 2016 | 51.002 | 4.866 |
| *Vespula vulgaris* | BEL25B6 | E of Mechelen | Belgium | 2016 | 51.014 | 4.639 |
| *Vespula vulgaris* | BEL27 | Kampenhout | Belgium | 27-Sep-11 | 50.920 | 4.563 |
| *Vespula vulgaris* | FR112 |  | France |  |  |  |
| *Vespula vulgaris* | FR113 |  | France |  |  |  |
| *Vespula vulgaris* | FR114 |  | France |  |  |  |
| *Vespula vulgaris* | FR115 | Gers | France |  | 43.637 | 0.450 |
| *Vespula vulgaris* | FR116 | Verna | France | 9-Sep-12 | 48.865 | 2.349 |
| *Vespula vulgaris* | GER345 | Staudt | Germany | 23-May-17 | 50.461 | 7.832 |
| *Vespula vulgaris* | GER354 | Staudt | Germany | 16-Jul-17 | 50.461 | 7.832 |
| *Vespula vulgaris* | GER355 | Staudt | Germany | 16-Jul-17 | 50.461 | 7.832 |
| *Vespula vulgaris* | GER357 | Koeppel | Germany | 26-Jun-17 | 50.429 | 7.751 |
| *Vespula vulgaris* | NZ2 | Little River, Canterbury | New Zealand | 23-Feb-18 | -43.761 | 172.807 |
| *Vespula vulgaris* | NZ3 | Little River, Canterbury | New Zealand | 23-Feb-18 | -43.760 | 172.808 |
| *Vespula vulgaris* | NZ30 | Rotokakahi Lake, Rotorua | New Zealand | 8-Apr-17 | -38.204 | 176.342 |
| *Vespula vulgaris* | NZ31 | Waitakere ranges, Auckland | New Zealand | 10-Apr-17 | -36.954 | 174.474 |
| *Vespula vulgaris* | NZ32 | Auckland University, Tamaki Campus | New Zealand |  | -36.852 | 174.769 |
| *Vespula vulgaris* | NZ33 | Auckland E. Ridge Shopping Centre | New Zealand |  | -36.852 | 174.769 |
| *Vespula vulgaris* | NZ34 | Arthur's Pass, nest B | New Zealand | 7-Feb-12 | -42.983 | 171.733 |
| *Vespula vulgaris* | NZ35 | Arthur's Pass, nest C | New Zealand | 7-Feb-12 | -42.983 | 171.733 |
| *Vespula vulgaris* | NZ36 | Arthur's Pass, nest D | New Zealand | 7-Feb-12 | -42.983 | 171.733 |
| *Vespula vulgaris* | NZ37 | Arthur's Pass, nest E | New Zealand | 7-Feb-12 | -42.983 | 171.733 |
| *Vespula vulgaris* | NZ38 | Six Mile, Nelson. Nest 1, 2016 | New Zealand | 2016 | -41.769 | 172.957 |
| *Vespula vulgaris* | NZ39 | Tin Line, Nelson. Nest 1, 2014 | New Zealand | 2014 | -41.281 | 173.509 |
| *Vespula vulgaris* | NZ40 | Belmont, Wellington | New Zealand | 18-Apr-12 | -41.194 | 174.923 |
| *Vespula vulgaris* | NZ41 | 321 Willis Street, Te Aro, Wellington, nest 1 | New Zealand | 2013 | -41.296 | 174.771 |
| *Vespula vulgaris* | NZ42 | Breamhead, Whangarei | New Zealand | 20-Apr-12 | -35.725 | 174.324 |
| *Vespula vulgaris* | NZ43 | 15 Kotare Rd, Eastbourne, Wellington | New Zealand | Feb-13 | -41.292 | 174.898 |
| *Vespula vulgaris* | NZ44 | Castlepoint | New Zealand | 17-Feb-13 | -40.898 | 176.220 |
| *Vespula vulgaris* | NZ45 | 480 Leitn St., Dunedin | New Zealand | 1-Mar-13 | -45.879 | 170.503 |
| *Vespula vulgaris* | NZ46 | Braeburn, Nelson. Nest 3, 2016 | New Zealand | 2016 | -41.795 | 172.516 |
| *Vespula vulgaris* | NZ47 | Tokaanu, Taupo | New Zealand | 23-Feb-12 | -38.683 | 176.083 |
| *Vespula vulgaris* | NZ90 | Waitakere ranges, Auckland | New Zealand | 10-Apr-17 | -36.954 | 174.474 |
| *Vespula vulgaris* | NZ93 | Great Mercury Island | New Zealand | 3-Mar-17 | -36.631 | 175.818 |
| *Vespula vulgaris* | NZ123 | Waiheke Island | New Zealand | 16-Mar-17 | -36.802 | 175.108 |
| *Vespula vulgaris* | NZ139 | Devonport, Auckland | New Zealand | 7-Apr-17 | -36.832 | 174.798 |
| *Vespula vulgaris* | NZ140 | Devonport, Auckland | New Zealand | 7-Apr-17 | -36.832 | 174.798 |
| *Vespula vulgaris* | NZ141 | Mangawhai | New Zealand | 2-May-17 | -36.100 | 174.585 |
| *Vespula vulgaris* | NZ142 | Mangawhai | New Zealand | 2-May-17 | -36.100 | 174.585 |
| *Vespula vulgaris* | NZ168 | Buller river | New Zealand | 9-Feb-19 | -41.702 | 172.657 |
| *Vespula vulgaris* | NZ169 | Buller river | New Zealand | 9-Feb-19 | -41.702 | 172.657 |
| *Vespula vulgaris* | NZ170 | Hamner Springs | New Zealand | 9-Feb-19 | -42.525 | 172.810 |
| *Vespula vulgaris* | NZ171 | Hamner Springs | New Zealand | 9-Feb-19 | -42.525 | 172.810 |
| *Vespula vulgaris* | NZ172 | Murchison | New Zealand | 9-Feb-19 | -41.984 | 172.198 |
| *Vespula vulgaris* | NZ173 | Murchison | New Zealand | 9-Feb-19 | -41.984 | 172.198 |
| *Vespula vulgaris* | NZ174 | Picton | New Zealand | 9-Feb-19 | -41.294 | 174.014 |
| *Vespula vulgaris* | NZ198 | Hostel in Tairua | New Zealand | 22-Mar-17 | -37.005 | 175.848 |
| *Vespula vulgaris* | NZ314 | Slipper Island | New Zealand | 21-Mar-17 | -37.049 | 175.950 |
| *Vespula vulgaris* | NZ340 | Nest View Road, Waiheke Island | New Zealand | 15-Mar-17 | -36.784 | 175.055 |
| *Vespula vulgaris* | NZ401 | Buxton Ave., Karori, Wellington | New Zealand | 12-Dec-17 | -41.285 | 174.717 |
| *Vespula vulgaris* | NZ2T6 | Tin Line, Nelson | New Zealand | 2016 | -41.281 | 173.509 |
| *Vespula vulgaris* | NZGMI2 | Great Mercury Island | New Zealand | 3-Mar-17 | -36.629 | 175.818 |
| *Vespula vulgaris* | NZKAR2 | Buxton Ave., Karori, Wellington | New Zealand | 9-Jul-18 | -41.285 | 174.717 |
| *Vespula vulgaris* | NZ3S6 | Six Mile, Nelson | New Zealand | 2016 | -41.769 | 172.957 |
| *Vespula vulgaris* | NZ6B5 | Braeburn, Nelson | New Zealand | 2015 | -41.795 | 172.516 |
| *Vespula vulgaris* | RU48 | Moscow district | Russia | 17-Aug-12 | 55.215 | 37.913 |
| *Vespula vulgaris* | RU49 | Moscow district | Russia | 17-Aug-12 | 55.215 | 37.913 |
| *Vespula vulgaris* | RU50 | Moscow district | Russia | 11-Sep-12 | 55.215 | 37.913 |
| *Vespula vulgaris* | RU51 | Moscow district | Russia | 11-Sep-12 | 55.215 | 37.913 |
| *Vespula vulgaris* | RU52 | Moscow district | Russia | 11-Sep-12 | 55.215 | 37.913 |
| *Vespula vulgaris* | RU53 | Moscow district | Russia | 12-Sep-12 | 55.215 | 37.913 |
| *Vespula vulgaris* | RU54 | Moscow district | Russia | 17-Aug-12 | 55.215 | 37.913 |
| *Vespula vulgaris* | RU55 | Moscow district | Russia | 17-Aug-12 | 55.215 | 37.913 |
| *Vespula vulgaris* | RU56 | Moscow district | Russia | 17-Aug-12 | 55.215 | 37.913 |
| *Vespula vulgaris* | RU57 | Moscow district | Russia | 24-Aug-12 | 55.216 | 37.894 |
| *Vespula vulgaris* | RU58 | Moscow district | Russia | 24-Aug-12 | 55.216 | 37.911 |
| *Vespula vulgaris* | RU59 | Moscow district | Russia | 17-Aug-12 | 55.210 | 37.918 |
| *Vespula vulgaris* | RU60 | Moscow district | Russia | 11-Sep-12 | 55.216 | 37.911 |
| *Vespula vulgaris* | RU61 | Moscow district | Russia | 19-Aug-12 | 55.205 | 37.921 |
| *Vespula vulgaris* | RU62 | Moscow district | Russia | 19-Aug-12 | 55.205 | 37.921 |
| *Vespula vulgaris* | RU63 | Moscow district | Russia | 24-Aug-12 | 55.215 | 37.913 |
| *Vespula vulgaris* | RU64 | Moscow district | Russia | 17-Aug-12 | 55.215 | 37.913 |
| *Vespula vulgaris* | RU65 | Moscow district | Russia | 13-Sep-12 | 55.215 | 37.913 |
| *Vespula vulgaris* | RU66 | Moscow district | Russia | 11-Sep-12 | 55.215 | 37.913 |
| *Vespula vulgaris* | RU67 | Moscow district | Russia | 11-Sep-12 | 55.215 | 37.913 |
| *Vespula vulgaris* | SP103 | Lecinana, Burgos, 675 m | Spain | 23-Sep-12 | 42.341 | -3.704 |
| *Vespula vulgaris* | SP104 | Rio Ligros, Jabaloyas, Teruel, 1250 m | Spain | 6-Oct-12 | 42.724 | 0.284 |
| *Vespula vulgaris* | SP105 | Banos de Panticosa, Huesca, 1630 m | Spain | 16-Sep-12 | 42.724 | 0.284 |
| *Vespula vulgaris* | SP106 | Rabanal de los Caballeros, Palencia, 1040 m | Spain | 10-Aug-12 | 42.896 | -4.475 |
| *Vespula vulgaris* | SP108 | Santa Maria de Redondo, Palencia, 1300 m | Spain | 13-Aug-12 | 42.990 | -4.433 |
| *Vespula vulgaris* | SP109 | Albarracin, Teruel, 1130 m | Spain | 6-Oct-12 | 40.346 | -1.106 |
| *Vespula vulgaris* | SP110 | Concejero, Burgos, 385 m | Spain | 23-Sep-12 | 43.103 | -3.349 |
| *Vespula vulgaris* | SWE1 | Kalmar | Sweden | 16-Aug-12 | 56.663 | 16.357 |
| *Vespula vulgaris* | SWE4 | Stockholm | Sweden | 14-Aug-12 | 59.260 | 17.994 |
| *Vespula vulgaris* | UK45 |  | United Kingdom | |  |  |
| *Vespula vulgaris* | UK47 |  | United Kingdom | |  |  |
| *Vespula vulgaris* | UK73 | Silwood Park, under conifer | United Kingdom | 4-Sep-18 | 51.410 | -0.642 |
| *Vespula vulgaris* | UK74 | Silwood Park, | United Kingdom | 6-Sep-18 | 51.410 | -0.643 |
| *Vespula vulgaris* | UK77 | Wallingford | United Kingdom | 14-Sep-18 | 51.590 | -1.119 |
| *Vespula vulgaris* | UK78 | Silwood Park | United Kingdom | 17-Sep-18 | 51.408 | -0.643 |
| *Vespula vulgaris* | UK79 | Eghan, CABI | United Kingdom | 18-Sep-18 | 51.418 | -0.569 |
| *Vespula vulgaris* | UK80 | Morden Hall, Happy Valley | United Kingdom | 19-Sep-18 | 51.395 | -0.177 |
| *Vespula vulgaris* | UK81 | Morden Hall, waterways#2 | United Kingdom | 19-Sep-18 | 51.395 | -0.170 |
| *Vespula vulgaris* | UK82 | Morden Hall, waterways#1 | United Kingdom | 19-Sep-18 | 51.395 | -0.170 |
| *Vespula vulgaris* | UK83 | Morden Hall Café | United Kingdom | 19-Sep-18 | 51.403 | -0.189 |
| *Vespula vulgaris* | UK84 | Silwood Park by dormitories | United Kingdom | 24-Sep-18 | 51.410 | -0.640 |
| *Vespula vulgaris* | UK86 | Reagent’s Park, London | United Kingdom | 27-Sep-18 | 51.530 | -0.159 |
| *Vespula vulgaris* | UK88 | Eghan, CABI | United Kingdom | 1-Sep-16 | 51.419 | -0.568 |
| *Vespula vulgaris* | UK146 | Ash Hill, Berkshire | United Kingdom | 5-Oct-16 | 51.420 | -1.264 |
| *Vespula vulgaris* | UK148 | Park Estate, Devon | United Kingdom | 27-Sep-16 | 50.769 | -3.199 |
| *Vespula vulgaris* | UK149 | Winkworth Arboretum, Godalming, Surrey | United Kingdom | 22-Sep-16 | 51.164 | -0.583 |
| *Vespula vulgaris* | UK150 | Primley Road, Sidmouth, Devon | United Kingdom | 27-Sep-16 | 50.695 | -3.233 |
| *Vespula vulgaris* | UK152 | Oxborough Road, West Norfolk | United Kingdom | | 52.581 | 0.569 |
| *Vespula vulgaris* | UK153 | Petworth small island, West Sussex | United Kingdom | 19-Sep-16 | 50.986 | -0.613 |
| *Vespula vulgaris* | UK154 | Sussex | United Kingdom | 20-Sep-16 | 50.999 | 0.013 |
| *Vespula vulgaris* | UK155 | Sussex | United Kingdom | 20-Sep-16 | 50.999 | 0.013 |
| *Vespula vulgaris* | UK156 | West Norfolk | United Kingdom | 23-Sep-16 | 52.581 | 0.569 |
| *Vespula vulgaris* | UK157 | Sussex | United Kingdom | | 50.988 | -0.611 |
| *Vespula vulgaris* | UK158 | Arnos Vale, Bristol | United Kingdom | 18-Sep-17 | 51.441 | -2.565 |
| *Vespula vulgaris* | UK159 | Dorchester | United Kingdom | 28-Oct-17 | 50.707 | -2.439 |
| *Vespula vulgaris* | UK160 | Poole | United Kingdom | 28-Oct-17 | 50.739 | -1.919 |
| *Vespula vulgaris* | UK161 | Park Estate, Devon | United Kingdom | 27-Sep-16 | 50.769 | -3.199 |
| *Apis mellifera* | AM-1 |  | New Zealand | 2015 |  |  |
| *Bombus terrestris* | BT-19-N1W1 | | New Zealand | 2015 |  |  |
| *Polistes dominula* | PD-B | Budge Street, Blenheim, New Zealand | New Zealand | 14-Apr-16 | -41.505 | 173.968 |
| *V. germanica* | AU124 | Zentralfriedhof, Graz | Austria | 9-Oct-18 | 47.045 | 15.424 |
| *V. maculifrons* | US120 | Statesboro, Georgia | United States | 12-Nov-18 | 32.418 | -81.787 |
| *V. pensylvanica* | HAW191 | Hawaii Volcanoes National Park, Hawaii’s Big Island | United States |  | 19.422 | -155.203 |

**Table S2.** List of the genes predicted to be associated with spermatogenesis showing genetic variation, number of individuals sequenced for each gene and relative position (p) of substitutions. NZ = New Zealand, UK = United Kingdom.

| Gene target | Length sequenced product (bp) | # variable sites | Positions with single nucleotide substitutions (bp) | Positions with insertions or deletions | Individuals screened (*n*) | How many individuals presented the substitutions? Where were they from? | Reference indicating the genes were associated with spermatogenesis |
| --- | --- | --- | --- | --- | --- | --- | --- |
| **Boule protein, region 1** | 719 | 1 | 576 | 0 | 125 | 1 wasp (France) | Mikhaylova et al. (2006) Transcriptional regulation by Modulo integrates meiosis and spermatid differentiation in male germ line. PNAS 103: 11975-11980 |
| **Boule protein, region 2** | 521 | 1 | 428 | 0 | 32 | 2 wasps (1 Belgium, 1 Sweden) | Sekiné et al. (2015) The boule gene is essential for spermatogenesis of haploid insect male. Developmental Biology 399: 154–163 |
| **cell division cycle 25, region A** | 576 | 3 | 168, 411, 424 | 0 | 32 | p168: 4 NZ wasps; p411: 3 wasps (1 NZ, 1 Belgium, 1 Sweden); | Sekiné et al. (2015) The boule gene is essential for spermatogenesis of haploid insect male. Developmental Biology 399: 154–163 |
|  |  |  |  |  |  | p424: 5 wasps (2 NZ, 1 Belgium, 1 Germany, 1 Sweden) |  |
| **cell division cycle 25, region B** | 294 | 1 | 257 | 0 | 123 | 43 wasps (3 Austria, 3 Belgium, 4 France, 1 Germany, 4 NZ, 7 Russia, 5 Spain, 1 Sweden, 15 UK) | Sekiné et al. (2015) The boule gene is essential for spermatogenesis of haploid insect male. Developmental Biology 399: 154–163 |
| **DNA helicase MCM8-like (MCM8)** | 309 | 0 | 0 | 0 | 29 | – | Ferree et al. (2015) Identification of Genes Uniquely Expressed in the Germ-Line Tissues of the Jewel Wasp *Nasonia vitripennis*. G3: Genes, Genomes & Genetics 5: 2647–2653. |
| **Fuzzy onions (fzo)** | 597 | 2 | 504, 573 | 0 | 30 | p504: 13 wasps (9 NZ, 3 Belgium, 1 Germany); | Kandul et al. (2019) Transforming insect population control with precision guided sterile males with demonstration in flies. Nature Communications 10: 84. |
|  |  |  |  |  |  | p573: 7 wasps (3 Belgium, 2 NZ, 1 UK, 1 Germany) |  |
| **Ocnus (ocn)** | 939 | 6 | 98, 266, 369, 474, 610, 694 | 0 | 123 | p98: 5 wasps (3 Russia, 1 Spain, 1 UK); | Parsch et al. (2001) Molecular evolution of the ocnus and janus genes in the *Drosophila melanogaster* species subgroup. Molecular Biology and Evolution 18: 801–811. |
|  |  |  |  |  |  | p266: 4 wasps (3 Russia, 1 Spain); |  |
|  |  |  |  |  |  | p369: 46 wasps (2 Belgium, 3 Germany, 19 NZ, 3 Spain, 1 Sweden, 15 UK, 3 Russia); |  |
|  |  |  |  |  |  | p474: 8 wasps (1 Belgium, 6 NZ, 1 Sweden); |  |
|  |  |  |  |  |  | p610: 9 wasps (2 NZ, 3 Spain, 2 UK, 2 Russia); |  |
|  |  |  |  |  |  | p694: 16 wasps (3 France, 5 NZ, 4 Spain, 3 UK, 1 Russia). |  |
| **Sperm-specific dynein intermediate chain (sdic)** | 850 | 10 | 30, 92, 169, 501, 506, 609, 700, 719, 801. | 1: p530-536, 6 bp (TCAATC) deletion in 29 individuals (12 Russian, 11 NZ, 2 Austria, 2 Sweden, 2 UK) | 122 | p30: 1 Russian wasp; | Parsch et al. (2001) Molecular evolution of the ocnus and janus genes in the *Drosophila melanogaster* species subgroup. Molecular Biology and Evolution 18: 801–811. |
|  |  |  |  |  |  | p92: 4 wasps (3 NZ, 1 UK); |  |
|  |  |  |  |  |  | p169: 1 Russian wasp; |  |
|  |  |  |  |  |  | p501: 1 Russian wasp; |  |
|  |  |  |  |  |  | p506: 4 wasps (1 France, 2 NZ, 1 Russia) |  |
|  |  |  |  |  |  | p609: 25 wasps (12 Russia, 7 NZ, 4 UK, 2 Austria); |  |
|  |  |  |  |  |  | p700: 23 wasps (12 Russia, 8 NZ, 1 UK, 2 Austria); |  |
|  |  |  |  |  |  | p719: 7 wasps (1 Russia, 5 NZ, 1 Austria); |  |
|  |  |  |  |  |  | p801: 24 wasps (11 Russia, 8 NZ, 3 UK, 2 Austria). |  |

**Table S3.** The sgRNA sequences designed to target *Vespula vulgaris* spermatogenesis genes. Protospacer adjacent motif (PAM) sequences are indicated in red and are 3 base pair DNA sequences immediately following the DNA sequence targeted by the Cas9 nuclease.

| **sgRNA name** | **Sequence** | **Notes** |
| --- | --- | --- |
| *boule v1* | GCTGGAGTTTCAAAAGGTTACGG | Designed to target all samples |
|  |  |  |
| *sdic v1* | AGATTCAATCAGTTATGAAGTGG | Designed to target samples **without** 6bp indel (indel negative) |
|  |  |  |
| *sdic v2* | CAATCAAGATAGTTATGAAGTGG | Designed to target samples **with** 6bp indel (indel positive) |
|  |  |  |
| *ocnus v1* | AAAATTTTATGAACTGCTGCAGG | Designed to target samples with T at position 266. The sgRNA is to the reverse sequence |
|  |  |  |
| *ocnus v2* | AAAATTTTGTGAACTGCTGCAGG | Designed to target samples with C at position 266. The sgRNA is to the reverse sequence |
|  |  |  |

**Table S4:** Primer pairs developed for *Vespula vulgaris* in this study.

| **Gene name** | **Primer name** | **Primer sequence 5' to 3'end** | **PCR product length (bp)** |
| --- | --- | --- | --- |
| Boule protein, region 1 | Boule-P1-F | TGTCAAGTTTCGTGCCAAAATGA | 783 |
|  | Boule-P1-R | CCAGAACCACCATCGAACGA |  |
| Boule protein, region 2 | Boule-P2-F | TGCTGATCGTGCTGGAGTTT | 521 |
|  | Boule-P2-R | TGCCGCTGCATTGTAGAATG |  |
| Cell division cycle 25, region A | cdc25A-F2 | GCATTTTACCTCTTGCCGAG | 576 |
|  | cdc25A-R2 | GCCGTTCAATCCCGATAAGT |  |
| Cell division cycle 25, region B | cdc25B-F | GGAAATGTTTGTCTCTTCATTGCA | 294 |
|  | cdc25B-R | TGAAATAAAAGAGAACGGGAGAAAA | |
| Predicted DNA helicase MCM8-like | MCM8-F | CGAGATATTAAAGTGAATTCCTATGGT | 309 |
|  | MCM8-R | TGATGTATTGGGAAAAGGGAGAT | |
| Fuzzy onions | fzo-F | AGCACTGCAGAATGATCGCA | 597 |
|  | fzo-R | TCAGAAGCAGATGCATCCCA |  |
| Ocnus | ocn-F | ACGACAAGAGTTAACAATTCTTCGT | 1042 |
|  | ocn-R | CGGAAGTTCCAAAGAAAAGTTATGG | |
| Sperm-specific dynein intermediate chain | sdic-F | TACCGGTACAATGGATGGCG | 951 |
|  | sdic-R | AAGCTAAGCAAGTAGCCGCT |  |
|  |  |  |  |

**Table S5:** GenBank accession numbers for each gene region and wasp and bee individuals in this study. x means that this individual was not sequenced for that locus. Not submitted = Sequence has not been submitted to GenBank but it is available from the authors upon request.

| **Species** | **Individual sample ID** | **boule, region 1** | **boule, region 2** | **cell division cycle 25, A** | **cell division cycle 25, B** | **Predicted DNA helicase MCM8-like** | **Ocnus** | **Sperm-specific dynein intermediate chain** | **Fuzzy onions** |
| --- | --- | --- | --- | --- | --- | --- | --- | --- | --- |
| *Vespula vulgaris* | AU125 | MN088861 | x | x | MN089050 | x | MN089231 | MN089354 | x |
| *Vespula vulgaris* | AU126 | MN088862 | x | x | MN089051 | x | MN089232 | MN089355 | x |
| *Vespula vulgaris* | AU127 | MN088863 | x | x | MN089052 | x | MN089233 | MN089356 | x |
| *Vespula vulgaris* | AU131 | MN088864 | x | x | MN089053 | x | MN089234 | MN089444 | x |
| *Vespula vulgaris* | AU132 | MN088865 | x | x | MN089054 | x | MN089235 | MN089357 | x |
| *Vespula vulgaris* | AU135 | MN088866 | x | x | MN089055 | x | MN089236 | MN089445 | x |
| *Vespula vulgaris* | BEL1L5 | MN088867 | MN088986 | MN089018 | MN089056 | MN089202 | MN089237 | MN089358 | MN089172 |
| *Vespula vulgaris* | BEL2L5 | MN088868 | MN088987 | MN089019 | x | MN089203 | MN089238 | MN089359 | MN089173 |
| *Vespula vulgaris* | BEL2H5 | MN088869 | MN088988 | x | x | x | x | x | x |
| *Vespula vulgaris* | BEL3 | MN088870 | MN088989 | MN089020 | MN089057 | MN089204 | MN089239 | MN089360 | MN089174 |
| *Vespula vulgaris* | BEL17B6 | MN088871 | MN088990 | MN089021 | MN089058 | MN089205 | MN089240 | MN089361 | MN089175 |
| *Vespula vulgaris* | BEL19B6 | MN088872 | MN088991 | MN089022 | MN089059 | MN089206 | MN089241 | MN089362 | MN089176 |
| *Vespula vulgaris* | BEL21B6 | MN088873 | MN088992 | MN089023 | MN089060 | MN089207 | MN089242 | MN089363 | MN089177 |
| *Vespula vulgaris* | BEL22B6 | MN088874 | MN088993 | MN089024 | MN089061 | MN089208 | MN089243 | MN089364 | MN089178 |
| *Vespula vulgaris* | BEL23B6 | MN088875 | MN088994 | MN089025 | MN089062 | MN089209 | MN089244 | MN089365 | MN089179 |
| *Vespula vulgaris* | BEL25B6 | MN088876 | MN088995 | MN089026 | MN089063 | MN089210 | MN089245 | MN089366 | MN089180 |
| *Vespula vulgaris* | BEL27 | MN088877 | MN088996 | MN089027 | MN089064 | MN089211 | MN089246 | MN089367 | MN089181 |
| *Vespula vulgaris* | FR112 | MN088878 | x | x | MN089065 | x | MN089247 | MN089368 | x |
| *Vespula vulgaris* | FR113 | MN088879 | x | x | MN089066 | x | MN089248 | MN089369 | x |
| *Vespula vulgaris* | FR114 | MN088880 | x | x | MN089067 | x | MN089249 | MN089370 | x |
| *Vespula vulgaris* | FR115 | MN088881 | x | x | MN089068 | x | MN089250 | MN089371 | x |
| *Vespula vulgaris* | FR116 | MN088882 | x | x | MN089069 | x | MN089251 | MN089372 | x |
| *Vespula vulgaris* | GER345 | MN088883 | MN088997 | MN089028 | MN089070 | MN089212 | MN089252 | MN089373 | MN089182 |
| *Vespula vulgaris* | GER354 | MN088884 | MN088998 | MN089029 | MN089071 | MN089213 | MN089253 | MN089374 | MN089183 |
| *Vespula vulgaris* | GER355 | MN088885 | MN088999 | MN089030 | MN089072 | MN089214 | MN089254 | MN089375 | MN089184 |
| *Vespula vulgaris* | GER357 | MN088886 | MN089000 | MN089031 | MN089073 | MN089215 | MN089255 | MN089376 | MN089185 |
| *Vespula vulgaris* | NZ2 | MN088887 | x | x | MN089074 | x | MN089256 | MN089377 | x |
| *Vespula vulgaris* | NZ3 | MN088888 | x | x | MN089075 | x | MN089257 | MN089469 | x |
| *Vespula vulgaris* | NZ30 | MN088889 | MN089001 | MN089032 | MN089076 | MN089216 | MN089258 | MN089454 | MN089186 |
| *Vespula vulgaris* | NZ31 | MN088890 | MN089002 | MN089033 | MN089077 | MN089217 | MN089259 | MN089378 | MN089187 |
| *Vespula vulgaris* | NZ32 | MN088891 | MN089003 | MN089034 | MN089078 | MN089218 | MN089260 | MN089379 | MN089188 |
| *Vespula vulgaris* | NZ33 | MN088892 | MN089004 | MN089035 | MN089079 | MN089219 | MN089261 | MN089380 | MN089189 |
| *Vespula vulgaris* | NZ34 | MN088893 | x | x | MN089080 | x | MN089262 | MN089381 | x |
| *Vespula vulgaris* | NZ35 | MN088894 | x | x | MN089081 | x | MN089263 | MN089446 | x |
| *Vespula vulgaris* | NZ36 | MN088895 | x | x | MN089082 | x | MN089264 | MN089382 | x |
| *Vespula vulgaris* | NZ37 | MN088896 | x | x | MN089083 | x | MN089265 | MN089447 | x |
| *Vespula vulgaris* | NZ38 | MN088897 | x | x | MN089084 | x | MN089266 | MN089448 | x |
| *Vespula vulgaris* | NZ39 | MN088898 | x | x | MN089085 | x | MN089267 | MN089383 | x |
| *Vespula vulgaris* | NZ40 | MN088899 | x | x | MN089086 | x | MN089268 | MN089384 | x |
| *Vespula vulgaris* | NZ41 | MN088900 | x | x | MN089087 | x | MN089269 | MN089385 | x |
| *Vespula vulgaris* | NZ42 | MN088901 | x | x | MN089088 | x | MN089270 | MN089386 | x |
| *Vespula vulgaris* | NZ43 | MN088902 | x | x | MN089089 | x | MN089271 | MN089449 | x |
| *Vespula vulgaris* | NZ44 | MN088903 | x | x | MN089090 | x | MN089272 | MN089450 | x |
| *Vespula vulgaris* | NZ45 | MN088904 | x | x | MN089091 | x | MN089273 | MN089387 | x |
| *Vespula vulgaris* | NZ46 | MN088905 | x | x | MN089092 | x | MN089274 | MN089388 | x |
| *Vespula vulgaris* | NZ47 | MN088906 | x | x | MN089093 | x | MN089275 | MN089451 | x |
| *Vespula vulgaris* | NZ90 | MN088907 | x | x | MN089094 | x | MN089276 | MN089389 | x |
| *Vespula vulgaris* | NZ93 | MN088908 | MN089005 | MN089036 | MN089095 | x | MN089277 | MN089390 | x |
| *Vespula vulgaris* | NZ123 | MN088909 | x | x | MN089096 | x | MN089278 | MN089391 | x |
| *Vespula vulgaris* | NZ139 | MN088910 | x | x | MN089097 | x | MN089279 | MN089392 | x |
| *Vespula vulgaris* | NZ140 | MN088911 | x | x | MN089098 | x | MN089280 | MN089393 | x |
| *Vespula vulgaris* | NZ141 | MN088912 | x | x | MN089099 | x | MN089281 | MN089394 | x |
| *Vespula vulgaris* | NZ142 | MN088913 | x | x | MN089100 | x | MN089282 | MN089395 | x |
| *Vespula vulgaris* | NZ168 | MN088914 | x | x | MN089101 | x | MN089283 | MN089452 | x |
| *Vespula vulgaris* | NZ169 | MN088915 | x | x | MN089102 | x | MN089284 | MN089396 | x |
| *Vespula vulgaris* | NZ170 | MN088916 | x | x | MN089103 | x | MN089285 | MN089397 | x |
| *Vespula vulgaris* | NZ171 | MN088917 | x | x | MN089104 | x | MN089286 | MN089398 | x |
| *Vespula vulgaris* | NZ172 | MN088918 | x | x | MN089105 | x | MN089287 | MN089399 | x |
| *Vespula vulgaris* | NZ173 | MN088919 | x | x | MN089106 | x | MN089288 | MN089470 | x |
| *Vespula vulgaris* | NZ174 | MN088920 | x | x | MN089107 | x | MN089289 | MN089453 | x |
| *Vespula vulgaris* | NZ198 | MN088921 | MN089006 | MN089037 | MN089108 | MN089220 | MN089290 | MN089400 | MN089190 |
| *Vespula vulgaris* | NZ314 | MN088922 | MN089007 | MN089038 | MN089109 | MN089221 | MN089291 | MN089401 | MN089191 |
| *Vespula vulgaris* | NZ340 | MN088923 | MN089008 | MN089039 | MN089110 | MN089222 | MN089292 | MN089402 | MN089192 |
| *Vespula vulgaris* | NZ401 | MN088924 | MN089009 | MN089040 | MN089111 | MN089223 | MN089293 | MN089403 | MN089193 |
| *Vespula vulgaris* | NZ2T6 | MN088925 | MN089011 | MN089041 | MN089112 | MN089224 | MN089294 | MN089404 | MN089194 |
| *Vespula vulgaris* | NZGMI2 | MN088926 | MN089010 | MN089042 | MN089113 | MN089225 | MN089295 | MN089405 | MN089195 |
| *Vespula vulgaris* | NZKAR2 | MN088927 | MN089012 | MN089043 | MN089114 | MN089226 | MN089296 | MN089406 | MN089196 |
| *Vespula vulgaris* | NZ3S6 | MN088928 | MN089013 | MN089044 | MN089115 | MN089227 | MN089297 | x | MN089197 |
| *Vespula vulgaris* | NZ6B5 | MN088929 | MN089014 | MN089045 | MN089116 | MN089228 | x | x | x |
| *Vespula vulgaris* | RU48 | MN088930 | x | x | MN089117 | x | MN089298 | MN089407 | x |
| *Vespula vulgaris* | RU49 | MN088931 | x | x | MN089118 | x | MN089299 | MN089408 | x |
| *Vespula vulgaris* | RU50 | MN088932 | x | x | MN089119 | x | MN089300 | MN089409 | x |
| *Vespula vulgaris* | RU51 | MN088933 | x | x | MN089120 | x | MN089301 | MN089410 | x |
| *Vespula vulgaris* | RU52 | MN088934 | x | x | MN089121 | x | MN089302 | MN089411 | x |
| *Vespula vulgaris* | RU53 | MN088935 | x | x | MN089122 | x | MN089303 | MN089455 | x |
| *Vespula vulgaris* | RU54 | MN088936 | x | x | MN089123 | x | MN089304 | MN089456 | x |
| *Vespula vulgaris* | RU55 | MN088937 | x | x | MN089124 | x | MN089305 | MN089412 | x |
| *Vespula vulgaris* | RU56 | MN088938 | x | x | MN089125 | x | MN089306 | MN089457 | x |
| *Vespula vulgaris* | RU57 | MN088939 | x | x | MN089126 | x | MN089307 | MN089458 | x |
| *Vespula vulgaris* | RU58 | MN088940 | x | x | MN089127 | x | MN089308 | MN089459 | x |
| *Vespula vulgaris* | RU59 | MN088941 | x | x | MN089128 | x | MN089309 | MN089460 | x |
| *Vespula vulgaris* | RU60 | MN088942 | x | x | MN089129 | x | MN089310 | MN089461 | x |
| *Vespula vulgaris* | RU61 | MN088943 | x | x | MN089130 | x | MN089311 | MN089436 | x |
| *Vespula vulgaris* | RU62 | MN088944 | x | x | MN089131 | x | MN089312 | MN089462 | x |
| *Vespula vulgaris* | RU63 | MN088945 | x | x | MN089132 | x | MN089313 | MN089463 | x |
| *Vespula vulgaris* | RU64 | MN088946 | x | x | MN089133 | x | MN089314 | MN089464 | x |
| *Vespula vulgaris* | RU65 | MN088947 | x | x | MN089134 | x | MN089315 | MN089465 | x |
| *Vespula vulgaris* | RU66 | MN088948 | x | x | MN089135 | x | MN089316 | MN089466 | x |
| *Vespula vulgaris* | RU67 | MN088949 | x | x | MN089136 | x | MN089317 | MN089435 | x |
| *Vespula vulgaris* | SP103 | MN088950 | x | x | MN089137 | x | MN089318 | MN089437 | x |
| *Vespula vulgaris* | SP104 | MN088951 | x | x | MN089138 | x | MN089319 | MN089438 | x |
| *Vespula vulgaris* | SP105 | MN088952 | x | x | MN089139 | x | MN089320 | MN089439 | x |
| *Vespula vulgaris* | SP106 | MN088953 | x | x | MN089140 | x | MN089321 | MN089440 | x |
| *Vespula vulgaris* | SP108 | MN088954 | x | x | MN089141 | x | MN089322 | MN089441 | x |
| *Vespula vulgaris* | SP109 | MN088955 | x | x | MN089142 | x | MN089323 | MN089442 | x |
| *Vespula vulgaris* | SP110 | MN088956 | x | x | MN089143 | x | MN089324 | MN089443 | x |
| *Vespula vulgaris* | SWE1 | MN088957 | MN089015 | MN089046 | MN089144 | MN089229 | MN089325 | MN089472 | MN089198 |
| *Vespula vulgaris* | SWE4 | MN088958 | MN089016 | MN089047 | MN089145 | MN089230 | MN089326 | MN089473 | MN089199 |
| *Vespula vulgaris* | UK45 | MN088959 | x | MN089048 | MN089146 | x | MN089327 | x | MN089200 |
| *Vespula vulgaris* | UK47 | MN088960 | MN089017 | MN089049 | MN089147 | x | MN089328 | not submitted | MN089201 |
| *Vespula vulgaris* | UK73 | MN088961 | x | x | MN089148 | x | MN089329 | MN089413 | x |
| *Vespula vulgaris* | UK74 | x | x | x | MN089149 | x | x | not submitted | x |
| *Vespula vulgaris* | UK77 | MN088962 | x | x | MN089150 | x | MN089330 | MN089414 | x |
| *Vespula vulgaris* | UK78 | MN088963 | x | x | x | x | MN089331 | MN089415 | x |
| *Vespula vulgaris* | UK79 | MN088964 | x | x | x | x | MN089332 | MN089416 | x |
| *Vespula vulgaris* | UK80 | MN088965 | x | x | MN089151 | x | MN089333 | MN089417 | x |
| *Vespula vulgaris* | UK81 | MN088966 | x | x | MN089152 | x | MN089334 | MN089418 | x |
| *Vespula vulgaris* | UK82 | MN088967 | x | x | MN089153 | x | MN089335 | MN089419 | x |
| *Vespula vulgaris* | UK83 | MN088968 | x | x | MN089154 | x | MN089336 | MN089420 | x |
| *Vespula vulgaris* | UK84 | MN088969 | x | x | MN089155 | x | MN089337 | MN089468 | x |
| *Vespula vulgaris* | UK86 | MN088970 | x | x | MN089156 | x | MN089338 | MN089421 | x |
| *Vespula vulgaris* | UK88 | MN088971 | x | x | MN089157 | x | MN089339 | MN089422 | x |
| *Vespula vulgaris* | UK146 | MN088972 | x | x | MN089158 | x | MN089340 | MN089423 | x |
| *Vespula vulgaris* | UK148 | MN088973 | x | x | MN089159 | x | MN089341 | MN089471 | x |
| *Vespula vulgaris* | UK149 | MN088974 | x | x | MN089160 | x | MN089342 | MN089424 | x |
| *Vespula vulgaris* | UK150 | MN088975 | x | x | MN089161 | x | MN089343 | MN089425 | x |
| *Vespula vulgaris* | UK152 | MN088976 | x | x | MN089162 | x | MN089344 | MN089426 | x |
| *Vespula vulgaris* | UK153 | MN088977 | x | x | MN089163 | x | MN089345 | MN089427 | x |
| *Vespula vulgaris* | UK154 | MN088978 | x | x | MN089164 | x | MN089346 | MN089428 | x |
| *Vespula vulgaris* | UK155 | MN088979 | x | x | MN089165 | x | MN089347 | MN089429 | x |
| *Vespula vulgaris* | UK156 | MN088980 | x | x | MN089166 | x | MN089348 | MN089430 | x |
| *Vespula vulgaris* | UK157 | MN088981 | x | x | MN089167 | x | MN089349 | MN089431 | x |
| *Vespula vulgaris* | UK158 | MN088982 | x | x | MN089168 | x | MN089350 | MN089432 | x |
| *Vespula vulgaris* | UK159 | MN088983 | x | x | MN089169 | x | MN089351 | MN089433 | x |
| *Vespula vulgaris* | UK160 | MN088984 | x | x | MN089170 | x | MN089352 | MN089434 | x |
| *Vespula vulgaris* | UK161 | MN088985 | x | x | MN089171 | x | MN089353 | MN089467 | x |
| *Apis mellifera* | AM-1 | not submitted | not submitted | not submitted | not submitted | not submitted | not submitted | not submitted | not submitted |
| *Bombus terrestris* | BT-19-N1W1 | not submitted | not submitted | not submitted | not submitted | not submitted | not submitted | not submitted | not submitted |
| *Polistes dominula* | PD-B | not submitted | not submitted | not submitted | not submitted | not submitted | not submitted | not submitted | not submitted |
| *V. germanica* | AU124 | not submitted | not submitted | not submitted | not submitted | not submitted | not submitted | not submitted | not submitted |
| *V. maculifrons* | US120 | not submitted | not submitted | not submitted | not submitted | not submitted | not submitted | not submitted | not submitted |
| *V. pensylvanica* | HAW | not submitted | not submitted | not submitted | not submitted | not submitted | not submitted | not submitted | not submitted |

**Table S6.** Primer pairs developed for the CRISPR-Cas9 assays on the non-target species: *Vespula germanica*, *Vespula pensylvanica*, *Polistes dominula*, *Bombus terrestris*, and *Apis mellifera* in this study. Only one primer pair was needed for each species as the duplicate sgRNA designs for *ocnus* and *sdic* were within the region covered by the primer pair.

| **Gene name** | **Species** | **Primer name** | **Primer sequence 5' to 3'end** | **PCR product length (bp)** | **Annealing temperature (deg C)** |
| --- | --- | --- | --- | --- | --- |
| *boule1* | *V. germanica* and *V. pensylvanica* | Boule-P1-F | TGTCAAGTTTCGTGCCAAAATGA | 783 | 64.7 |
|  |  | Boule-P1-R | CCAGAACCACCATCGAACGA |  |  |
|  | *Polistes dominula* | PD-Boule-FV2 | CTACTAGGCACAGAAGGTGGC | 953 | 64.1 |
|  |  | PD-Boule-RV2 | TGCTGAATATCCGTCCGACA |  |  |
|  | *Bombus terristris* | BT-Boule-1FV2 | GCATCAACGGGTGGTACTGA | 657 | 64.8 |
|  |  | BT-Boule-1R | CGTTGGTGTTGGAGCTGTTG |  |  |
| *ocnus* | *V. germanica* and *V. pensylvanica* | ocn-F | ACGACAAGAGTTAACAATTCTTCGT | 1042 | 62.4 |
|  |  | ocn-R | CGGAAGTTCCAAAGAAAAGTTATGG |  |  |
| *sdic* | *V. germanica* and *V. pensylvanica* | sdic-F | TACCGGTACAATGGATGGCG | 951 | 65 |
|  |  | sdic-R | AAGCTAAGCAAGTAGCCGCT |  |  |
|  | *Polistes dominula* | Polistes-sdic-Fv2 | GACGGCGAGGATGGATTGTA | 1289 | 64.7 |
|  |  | Polistes-sdic-R | GCTGGATGAGTTGGTGACCA |  |  |
|  | *Bombus terrestris* | Bombus-sdicFv3 | TGGTGAAGATGGAATGTGAGTAAAG | 1424 | 63.9 |
|  |  | Bombus-sdicR | TAGCGGTGGGTACTTCGGTA |  |  |
|  | *Apis mellifera* | Apis-sdic-FV2 | ACGATTCCTCGATCGTACCAG | 1213 | 64.3 |
|  |  | Apis-sdic-R | GAGTCATCTACAGCGGCGAA |  |  |

**Table S7.** Samples used in CRISPR-Cas9 cleavage assays. Twenty *V. vulgaris* individuals were selected, with ten from New Zealand and ten from Europe. In addition, five non-target species were selected (see Table S6). Gel IDs are the sample identification codes in the gel images (Supplementary Figures 1-6). For the twenty *V. vulgaris* individuals, sequencing of *sdic* revealed the presence or absence of a 6 bp deletion (*sdic* Del +/-) which was targeted with designed sgRNA (Supplementary Table S3). Similarly, sequencing of *ocnus* revealed a C or a T at position 266 (SNP 266 C/T), which was targeted with designed sgRNA (Supplementary Table S3). Specific location details for each of the samples are shown in Table S1, above.

| **Species** | **Gel ID** | **Sample ID** | **EU/NZ** | **Country** | ***sdic* Del +/-** | **ocnus SNP** |
| --- | --- | --- | --- | --- | --- | --- |
| *V. vulgaris* | 7 | NZ3S6 | NZ | New Zealand | Del - | 266 C |
| *V. vulgaris* | 13 | GER357 | EU | Germany | Del - | 266 C |
| *V. vulgaris* | 14 | BEL25B6 | EU | Belgium | Del - | 266 C |
| *V. vulgaris* | 30 | NZ30 | NZ | New Zealand | Del + | 266 C |
| *V. vulgaris* | 32 | NZ32 | NZ | New Zealand | Del - | 266 C |
| *V. vulgaris* | 35 | NZ35 | NZ | New Zealand | Del + | 266 C |
| *V. vulgaris* | 37 | NZ37 | NZ | New Zealand | Del + | 266 C |
| *V. vulgaris* | 38 | NZ38 | NZ | New Zealand | Del + | 266 C |
| *V. vulgaris* | 43 | NZ43 | NZ | New Zealand | Del + | 266 C |
| *V. vulgaris* | 45 | NZ45 | NZ | New Zealand | Del - | 266 C |
| *V. vulgaris* | 51 | RU51 | EU | Russia | Del - | 266 T |
| *V. vulgaris* | 62 | RU62 | EU | Russia | Del + | 266 T |
| *V. vulgaris* | 66 | RU66 | EU | Russia | Del + | 266 T |
| *V. vulgaris* | 82 | UK82 | EU | United Kingdom | Del - | 266 C |
| *V. vulgaris* | 103 | SP103 | EU | Spain | Del - | 266 C |
| *V. vulgaris* | 104 | SP104 | EU | Spain | Del - | 266 T |
| *V. vulgaris* | 112 | FR112 | EU | France | Del - | 266 C |
| *V. vulgaris* | 125 | AU125 | EU | Austria | Del - | 266 C |
| *V. vulgaris* | 139 | NZ139 | NZ | New Zealand | Del - | 266 C |
| *V. vulgaris* | 141 | NZ141 | NZ | New Zealand | Del - | 266 C |

**Fig. S1.** *Boule* sgRNA-Cas9 cleavage assay. Twenty *V. vulgaris* individuals were selected and a 783 bp region of the *boule* gene was amplified by PCR. PCR products were then digested with *boule* sgRNA-Cas9 and the digestion products resolved by electrophoresis in an agarose gel. An untreated sample was included as a reference. The sample numbers on the gel correspond to the Gel ID numbers in Supplementary Table S7, and EU/NZ indicates the samples were collected from either Europe or New Zealand. Where cleavage by *boule* sgRNA-Cas9 was expected, **Ex** is shown.


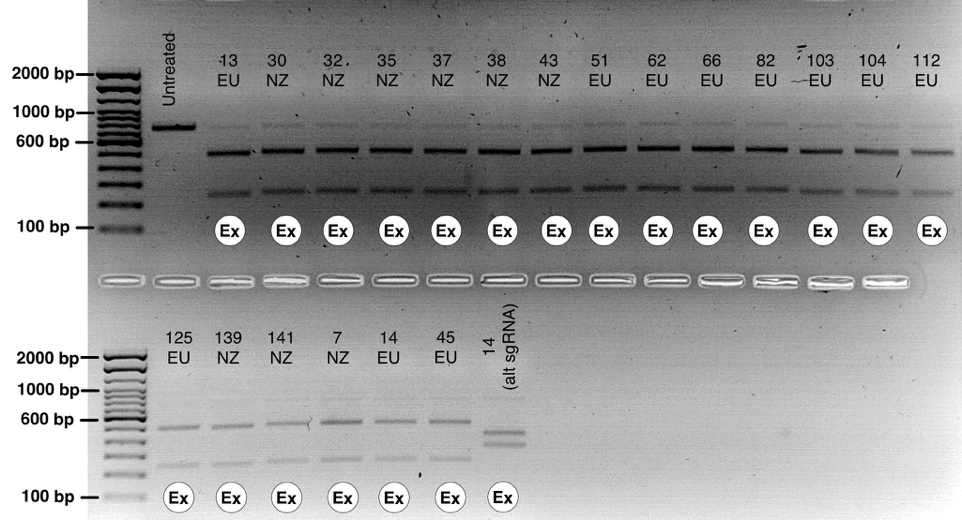


**Fig. S2.** *sdic* sgRNAv1-Cas9 cleavage assay. A 951 bp region of the *sdic* gene was PCR amplified from 20 *V. vulgaris* individuals. PCR products were then digested with *sdic* sgRNAv1-Cas9 and the digestion products resolved by electrophoresis in an agarose gel. The sample numbers on the gel correspond to the Gel ID numbers in Supplementary Table S1 & 7, and EU/NZ indicates the samples were collected from Europe or New Zealand. Where cleavage by *sdic* sgRNAv1-Cas9 was expected, **Ex** is shown, whereas unexpected results are indicated with **Un**.


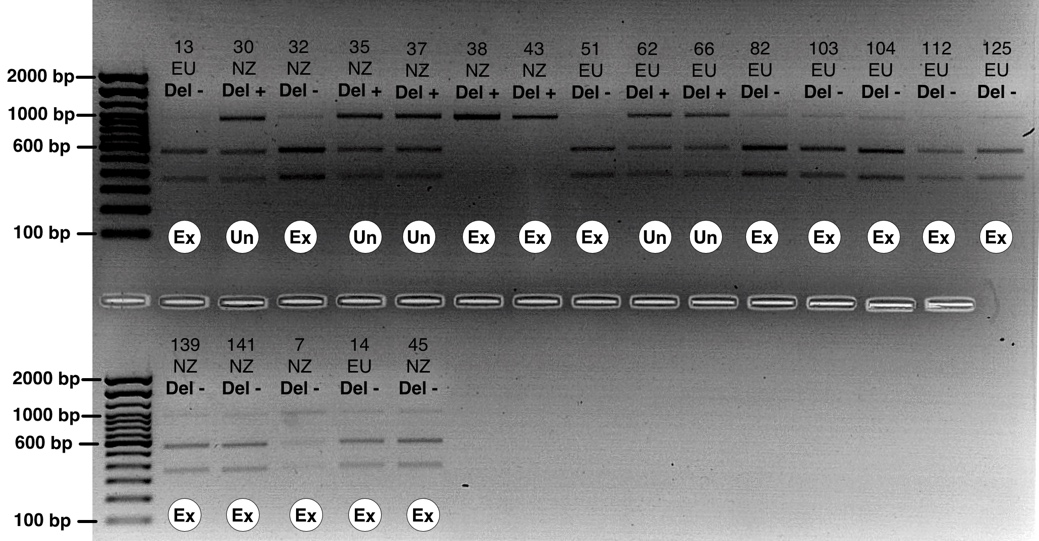


**Fig. S3.** *sdic* sgRNAv2-Cas9 cleavage assay. A 951 bp region of the *sdic* gene was PCR amplified from 20 *V. vulgaris* individuals. PCR products were then digested with *sdic* sgRNAv2-Cas9 and the digestion products resolved by electrophoresis in an agarose gel. An untreated sample was included as a reference. The sample numbers on the gel correspond to the Gel ID numbers in Supplementary Table S1 & 7, and EU/NZ indicates the samples were collected from Europe or New Zealand. Where cleavage by *sdic* sgRNAv2-Cas9 was expected, **Ex** is shown.


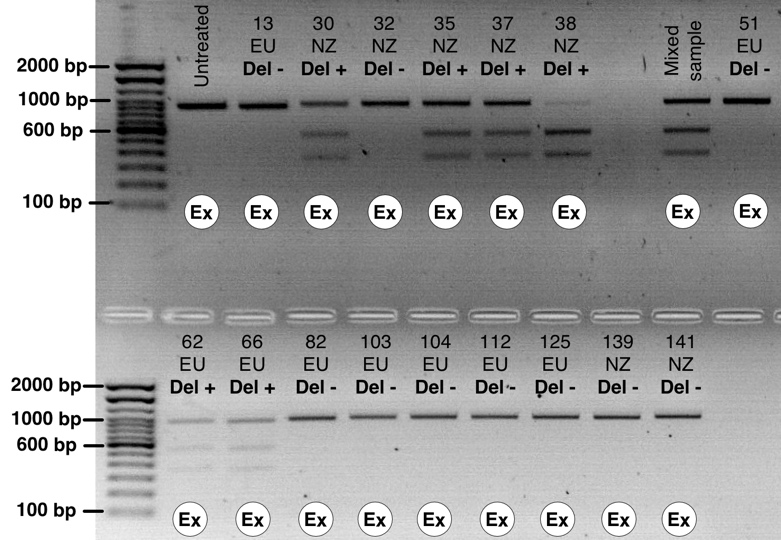


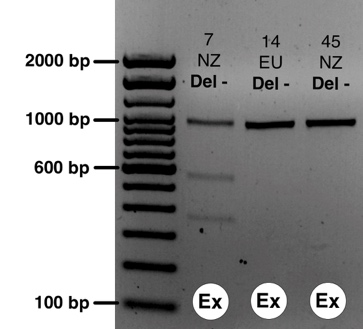


**Fig. S4.** *ocnus* sgRNAv1-Cas9 cleavage assay. A 1042 bp region of the *ocnus* gene was PCR amplified from 20 *V. vulgaris* individuals. PCR products were then digested with *ocnus* sgRNAv1-Cas9 and the digestion products resolved by electrophoresis in an agarose gel. An untreated sample was included as a reference. Sample numbers on the gel correspond to the Gel ID numbers in Supplementary Table S1 & 7, and EU/NZ indicates the samples were collected from Europe or New Zealand. The SNP identity is indicated with 266C/266T. Where cleavage by *ocnus* sgRNAv1 was expected, **Ex,** is shown.


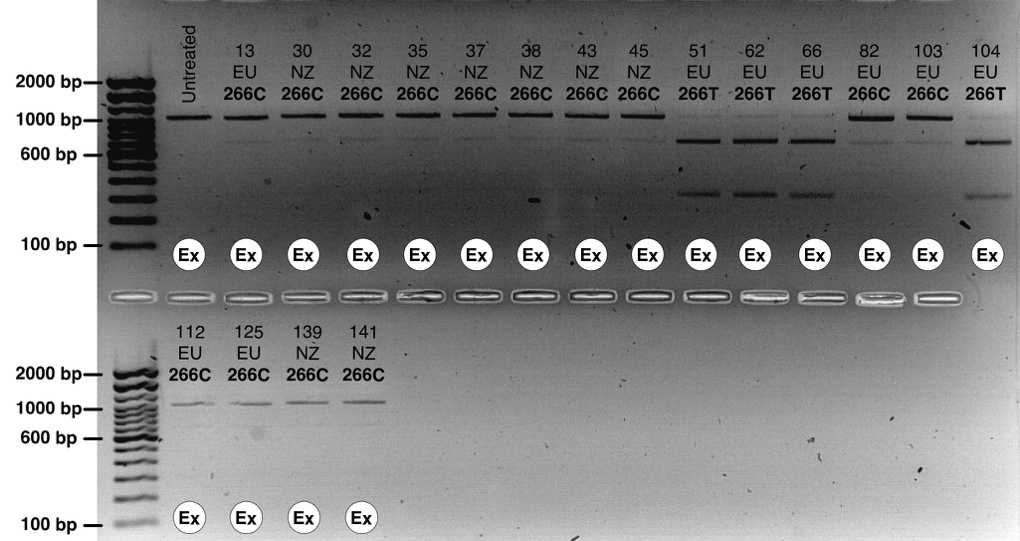


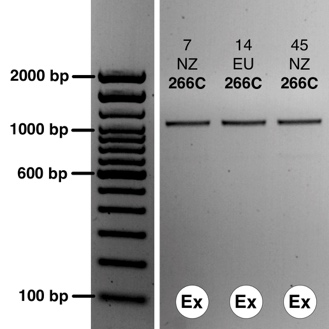


**Fig. S5.** *ocnus* sgRNAv2-Cas9 cleavage assay. A 1042 bp region of the *ocnus* gene was PCR amplified from 20 *V. vulgaris* individuals. PCR products were then digested with *ocnus* sgRNAv2-Cas9 and the digestion products resolved by electrophoresis in an agarose gel. An untreated sample was included as a reference. Sample numbers on the gel correspond to the Gel ID numbers in Supplementary Table S1 & 7, and EU/NZ indicates the samples were collected from Europe or New Zealand. The SNP identity is indicated with 266C/266T. Where cleavage by *ocnus* sgRNAv2 was expected, **Ex** is shown. whereas unexpected results are indicated with **Un**.


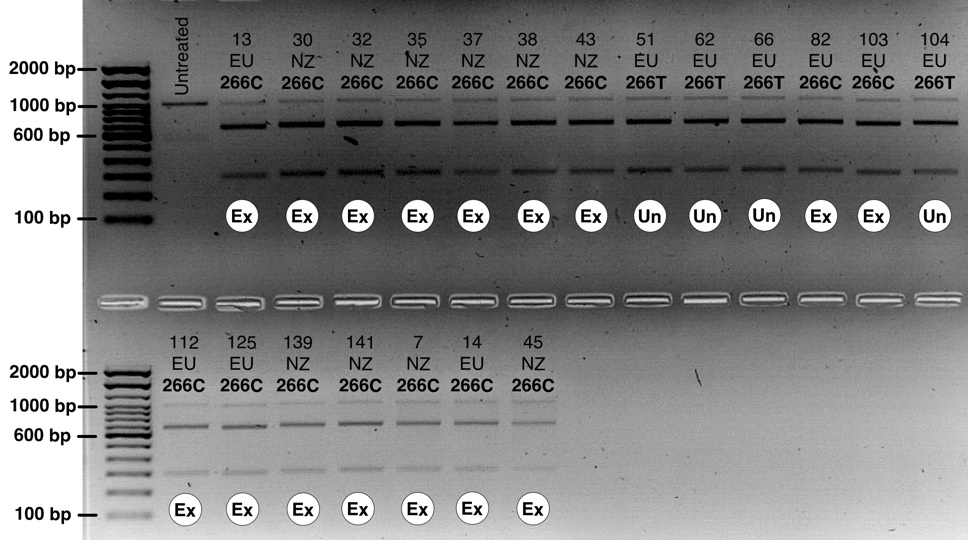


**Fig. S6.** Phylogenetic relatedness of the spermatogenesis gene *boule1*. The gene is relatively conserved within vespid wasps (including *Polistes dominula*), but distinct from other hymenopteran species and insects. Orthologs of the *boule1* gene were predicted with OrthoFinder (v. 2.3.1 with arguments*-M msa* -S diamond *-A mafft*) ^1^ using publicly available datasets from the Hymenopteran Genome Database ^2-13^. Species with more than 3 orthologs were trimmed to only 3 orthologs for visualization purposes using TreeGraph ^14^ to maintain correct phylogenetic distance. The corresponding ortholog tree was then stylized using FigTree v 1.4.4 (http://tree.bio.ed.ac.uk/software/figtree/). As noted in the main body of the manuscript, we struggled to find an ortholog in *Apis* to the boule gene. The genes included here were assigned to the orthogroup by OrthoFinder ^1^ but the highest only had a 31% amino acid sequence identity to the *Vespula vulgaris* boule protein in our analysis. Further, the BOULE/DAZ panther domain in the *Apis* proteins are incomplete, matching only 10 amino acids to the profile, while the *Vespula vulgaris* protein matches 379 amino acids to the PANTHER profile ^15^. This result may be due to the misannotation of this *Apis* protein, which contains several stop sequences in the published prediction.

**Fig. S7.** *boule*, *sdic* v1 and v2, *ocnus* v1 and v2 sgRNAv2-Cas9 cleavage assay on non-target species. A region of *boule* was amplified from *V. germanica* (VG, 783 bp), *V. pensylvanica* (VP, 783 bp), *Polistes dominula* (PD, 953 bp), and *Bombus terrestris* (BT, 657 bp), was PCR amplified and the products digested with *boule* sgRNA-Cas9. A region of *sdic* was amplified from *V. germanica* (951 bp), *V pensylvanica* (951 bp), *Polistes dominula* (1289 bp), *Bombus terrestris* (1424 bp), and *Apis mellifera* (AM, 1213 bp) was PCR amplified and the products digested with *sdic* sgRNA v1-Cas9 or *sdic* sgRNA v2-Cas9. A region of *ocnus* was amplified from *V. germanica* (1042 bp), and *V pensylvanica* (1042 bp) was PCR amplified and the products digested with *sdic* sgRNA v1-Cas9 or *sdic* sgRNA v2-Cas9. The digestion products were resolved by electrophoresis in an agarose gel. Where cleavage by sgRNA-Cas9 was expected, **Ex** is shown.


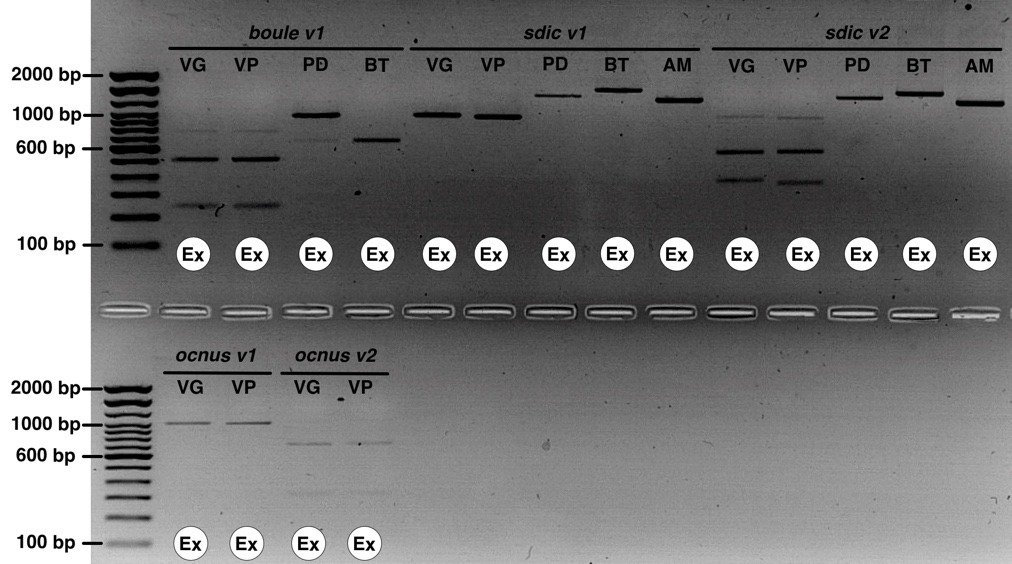


**Supplementary modelling methods and results**

We explore different scenarios for genetic control of haplo-diploid wasp populations, using a model for wasp population dynamics. The model is based on the seasonal biology of Vespulid wasps in New Zealand and simulates a homing endonuclease (HEG) gene drive for full or partial drone (male) sterility. We also implemented integer-based versions of each of the models, effectively adding demographic stochasticity and gene drift. The average results from the integer-based models conformed to the deterministic results so these models are not presented.

All models are based on the vespulid wasp life cycle shown in Figure S8. Diploid queens are univoltine, though there may be several cohorts of workers per year. Haploid males (drones) are produced in autumn and live only long enough to fertilise the new queens (gynes) produced at that time. Wasp nests are most obvious in summer when worker activity is at its peak, and this is typically when wasp populations are measured. Therefore, we take summer as the reference point of the model. Since the species we are concerned with have exactly one queen per nest, the queen densities correspond to summer nest densities.


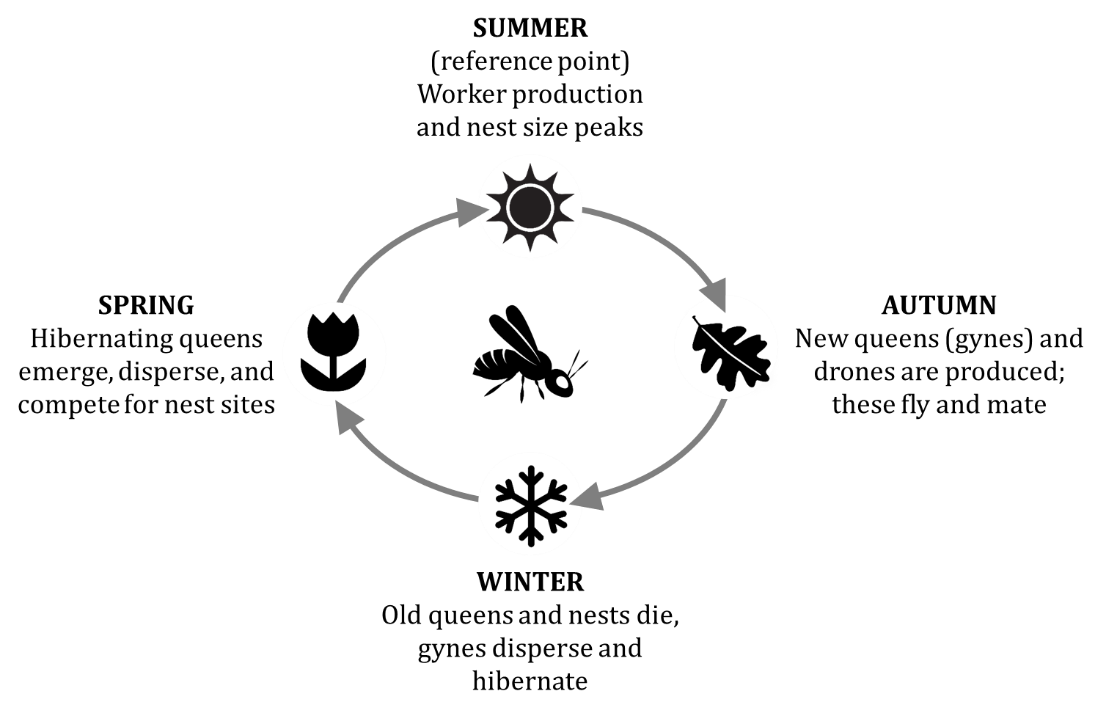


**Figure S8.** Summary of the lifecycles of *Vespula vulgaris* and *V. germanica* wasps in New Zealand.

To avoid confusion between generations, we refer to new generation queen wasps as “gynes” during autumn and winter. In the models, queens are denoted *Q,* gynes *G* and male drones *D*. For convenience we use the same symbols to indicate both the definition of the organism involved and also its density on the landscape; it should be clear from the context in which sense the symbols are being used. Subscripts indicate the alleles carried by each, either *w* for the wild type (WT) gene or *i* for the modified gene. We use a further subscript separated by a base line to also indicate the type of drone that a queen mated with; for example, *Q_wi_w_* indicates a heterozygous carrier queen that mated with a wild-type drone.

Soon after fertilization, a homing endonuclease gene (HEG) converts heterozygotes to homozygote carriers, resulting in the population consisting largely of homozygous wild type (WT) individuals and homozygous carriers. Heterozygous carriers may also be present due to imperfect homing of the drive.

At the reference time in summer the population may consist of queens of three relevant genotypes: wild type *Q_ww_*; heterozygous carriers *Q_wi_*; and homozygous carriers *Q_ii_*. Each summer queen was mated the previous autumn to either a wild type drone *D_w_* or a carrier drone *D_i_*. We need to track this because it affects the genotypes of the gynes and drones produced in the following autumn. Therefore, the summer population may be characterised by the densities of six queen types: *Q_ww_w_*, *Q_ww_i_*, *Q_wi_w_*, *Q_wi_i_*, *Q_ii_w_*, and *Q_ii_i_*.

The first step in the life cycle is the production of gynes and drones in autumn (Figure 1). Their genotypes depend on those of their mother queen and her mate as shown in Figure S9. Since we are interested in drone sterility, we use the parameter *p* to indicate the proportion of carrier drones that are sterile. The homing rate of the gene drive (proportion of heterozygotes transformed into homozygous carriers by the HEG) is denoted *h*.

**Figure S9**. Summary of the mating system of vespulid wasps with a HEG. The table entries show the results of the different mating combinations.

The mean potential number of gynes produced per WT queen in autumn is denoted *λ*. However, in some vespid species queen fecundity and nest size depend on the total number of viable sperm collected during mating ^16^. We define the effective sperm load carried by summer queens, *Z*•, to be the ratio of fertile sperm to total sperm collected at mating

$$Z\bullet=\frac{D_{w}+{c(1-p)D}_{i}}{D_{w}+{cD}_{i}}$$

where *c* is the relative mating competitiveness of carrier drones, *p* is the proportion of carrier drones that produce viable sperm. To avoid the use of additional subscripts, we use a dot (•) to indicate values in the following generation *t*+1. Hence sperm load potentially affects the number of progeny produced in the following generation. We then assume a power relationship between effective sperm load *Z* and realised fecundity *λZ^b^* (equivalent to nest size) as shown in Figure S10. When the sperm load fecundity factor *b* equals 0, there is no effect of sperm load on queen fecundity and nest size. However, as *b* becomes larger, sperm load increasingly constrains fecundity and nest size. We do not know what value for *b* might be appropriate for our target system, so we investigated a feasible range from 0 to 5. Note that the sperm load effect would differ from queen to queen depending on the success of the specific matings she was involved with. The way we have modelled it is a first approximation, but more detailed models would be needed if this mechanism was of primary interest.


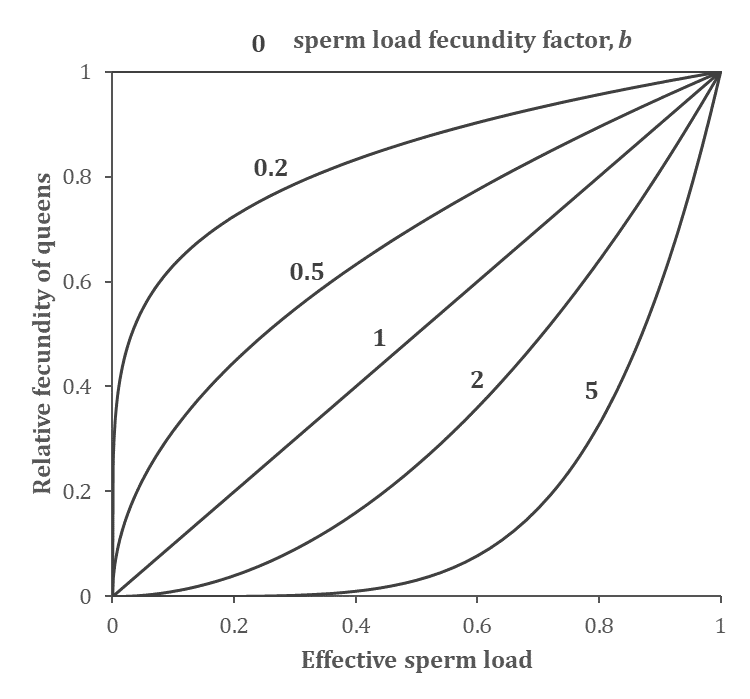


**Figure S10**. Assumed relationship between the effective sperm load, *Z*, and the relative fecundity of queen wasps, *Z^b^*, showing lines for different values of the sperm load fecundity factor *b*.

We assume that the number of drones produced from a nest is directly proportional to the production of gynes. The absolute number of drones produced is not important since we assume that there are sufficient for mating, and only the balance between WT and carrier drones affects the outcome from mating. Referring to Figure S9, we can tally the number of new-generation gynes *G* and drones *D* in autumn as:

$$G_{ww}=\lambda Z^{b}\left( Q_{ww\_w}+\frac{1}{2}Q_{wi\_w} \right)$$

$$G_{wi}=\lambda Z^{b}\left( \left( 1-p \right)\left( 1-h \right)Q_{ww\_i}+\frac{\left( 1-h \right)}{2}Q_{wi\_w}+\frac{\left( 1-p \right)\left( 1-h \right)}{2}Q_{wi\_i}+\left( 1-h \right)Q_{ii\_w} \right)$$

$$G_{ii}=\lambda Z^{b}\left( h\left( 1-p \right)Q_{ww\_i}+\frac{h}{2}Q_{wi\_w}+\frac{\left( 1-p \right)\left( 1+h \right)}{2}Q_{wi\_i}+hQ_{ii\_w}+\left( 1-p \right)Q_{ii\_i} \right)$$

$$D_{w}\propto Q_{ww\_w}+Q_{ww\_i}+\frac{1}{2}\left( Q_{wi\_w}+Q_{wi\_i} \right)$$

$$D_{i}\propto\frac{1}{2}\left( Q_{wi\_w}+Q_{wi\_i} \right)+Q_{ii\_w}+Q_{ii\_i}$$

These gynes and drones emerge from the hive to mix and mate (Figure S8). Gynes may mate several times with different drones ^17,18^ and we denote the mean number of mates per gyne as *m*. It is possible that the mating competitiveness of carrier drones may differ from that of WT drones, so we denote the relative competitiveness of carrier drones as *c*. Mating occurs away from the nests after considerable flight ^19^, so it is reasonable to assume the population is well mixed during mating. We also assume the population is large or self-contained, without significant immigration or emigration.

Drone sterility affects population density because unfertilised gynes, denoted *G*_0_, can produce only haploid drones. Importantly, these queens will contribute to the intense competition for nest sites that occurs in spring, potentially excluding fertile queens. However, after overwintering and competing for nest sites in spring, unfertilised queens are unable to produce diploid workers, so their nests fail to persist into summer.

To model this, we must tally the proportion of gynes that successfully receive sperm from at least one drone. If a proportion *p* of matings by carrier drones *D_i_* result in sperm transfer then the proportion of matings resulting no sperm transfer *pcD_i_* / (*D_w_* + *cD_i_*) and the probability that *m* matings will all fail to deliver viable sperm is closely approximated as [*pcD_i_* / (*D_w_* + *cD_i_*)]*^m^*. We assume that at least one fertile mating is sufficient for a gyne to later establish a successful nest. Therefore, the proportion of gynes receiving viable sperm over all matings, which we call the “fertilization rate” *f*, is:

$$f=1-\left( \frac{pcD_{i}}{D_{w}+cD_{i}} \right)^{m}$$

For the proportion *f* of gynes that do have at least one fertile mating we must then determine the genotype of the resulting offspring, noting that the outcomes may be affected by the biology of sperm handling during mating and fertilization. These details are as yet poorly understood for *V. vulgaris* and *V. germanica*, especially with respect to any HEG that interferes with normal sperm production. “Sterile” drones might produce sperm that are unable to fertilise the egg, or they might produce no sperm at all. Other social Hymenoptera display a range of scenarios for sperm competition and sperm clumping ^20^ leading to unequal contributions of different mates to egg fertilization.

For the purposes of modelling, sperm competition can be considered to be one of the factors leading to the relative competitiveness of carrier drones and included in the parameter *c*. If the mating population is large and well mixed, and if mating is random then sperm clumping does not affect the population-level outcomes for genotypes in the next generation: over all, mated queens will produce gyne genotypes in proportion to the type of drones present at mating. We let *j* denote the proportion of fertile matings that lead to the WT allele being passed on to offspring:

$$j=\frac{D_{w}}{D_{w}+c(1-p)D_{i}}$$

Now we can model the outcomes from mating, and the density of each type of gyne going into winter hibernation.

*G_ww_w_* = *f j* *G_ww_*

*G_ww_i_* = *f* (1– *j*) *G_ww_*

*G_wi_w_* = *f j* *G_wi_*

*G_wi_i_* = *f* (1– *j*) *G_wi_*

*G_ii_w_* = *f j* *G_ii_*

*G_ii_i_* = *f* (1– *j*) *G_ii_*

*G*_0_ = (1– *f*) (*G_ww_* + *G_wi_* + *G_ii_*)

The total population size in winter is the sum of these values, Σ*G*. A proportion *s* of these gynes will survive the winter to emerge as queens in spring, *s*Σ*G*.

We assume that intense spring competition between queens for nest sites is the major factor of density-dependent regulation in the wasp lifecycle ^21,22^. Applying the Beverton-Holt function for density-dependence, the proportion of competing queens securing nest sites is modelled as 1 / (1 + *N*/*n*), where *N* is the number of queens at that time and *n* is a nest site competition factor. For convenience we combine winter survival and spring nest competition into a single “survival factor” *g* where

$$g=\frac{s}{1+\frac{s\Sigma G}{n}}$$

Unfertilised gynes *G*_0_ fail to establish in spring. The remaining queens comprise the summer population. Therefore, the HEG model for one summer to the next (indicated by a dot •) is:

$$Q_{ww\_w}\bullet=\lambda Z^{b}fgj\left( Q_{ww\_w}+\frac{1}{2}Q_{wi\_w} \right)$$

$$Q_{wi\_w}\bullet=\lambda Z^{b}fgj\left( 1-h \right)\left( \left( 1-p \right)\left( Q_{ww\_i}+\frac{1}{2}Q_{wi\_i} \right)+\frac{1}{2}Q_{wi\_w}+Q_{ii\_w} \right)$$

$$Q_{ii\_w}\bullet=\lambda Z^{b}fgj\left( \left( 1-p \right)\left( {hQ}_{ww\_i}+\frac{\left( 1+h \right)}{2}Q_{wi\_i}+Q_{ii\_i} \right)+h\left( \frac{1}{2}Q_{wi\_w}+Q_{ii\_w} \right) \right)$$

$$Q_{ww\_i}\bullet=\frac{\left( 1-j \right)}{j}Q_{ww\_w}\bullet$$

$$Q_{wi\_i}\bullet=\frac{\left( 1-j \right)}{j}Q_{wi\_w}\bullet$$

$$Q_{ii\_i}\bullet=\frac{\left( 1-j \right)}{j}Q_{ii\_w}\bullet$$

where *f*, *j* and *g* are defined above.

We parameterised the model from values reported in the literature (Table S8). The nest site competition factor *n* determines the equilibrium nest density in summer, which we denote *K*. For a WT population without modified genes, *n* may be estimated as *K λs*/(*λs* – 1).

**Table S8.** Model parameters for vespulid wasps in New Zealand.

| **Parameter** | **Symbol** | **Default value** | **Source or range investigated** |
| --- | --- | --- | --- |
| *Wild type population parameters* | | | |
| gynes produced per WT nest | *λ* | 560 | Gruber et al. ^23^ |
| sperm load fecundity factor | *b* | 0 | 0 to 5 |
| mean number of matings per gyne | *m* | 2.5 | Dobelmann et al. ^18^ |
| overwintering survival | *s* | 0.02 | Archer ^24^ |
| summer nest equilibrium | *K* | 1350 /km^2^ | Lester et al. ^22^ |
| nest site competition factor | *n* | 1500 /km^2^ | Derived from *λ*, *s* and *K* |
| *Gene drive parameters* | | | |
| homing rate | *h* | 1 | 0 to 1 |
| relative mating competitiveness of carrier drones | *c* | 1 | 0 to 1 |
| proportion of carrier drones that are sterile | *p* | 1 | 0 to 1 |

*Case 1: Normal gene for drone sterility*

Here we assume that a nuclear gene is introduced that causes complete sterility of the drones that carry it (*p* = 1, hence *j* = 1), but without any gene drive (*h* = 0). The model simplifies to

*Q_ww_w_*• = *λZ^b^* *f* *g* (*Q_ww_w_* + *Q_wi_w_*/2)

*Q_wi_w_*• = *λZ^b^* *f* *g Q_wi_w_*/2

It is clear from these equations that WT queens can potentially increase faster than carrier queens, since half of the offspring from carriers are WT and only half are carriers. Hence carriers must decline in the population. Figure S11 shows the simulated effects of adding 100 carrier queens to the population every ten generations. Carriers of the sterility gene disappear from the population within a few generations of being introduced.

**
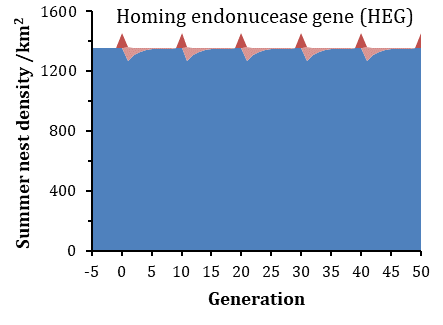
**

**Figure S11**. The effect of introducing 100 *Q_ii_w_* carrier queens /km^2^ into a wasp population once every ten years, where the introduced nuclear gene confers complete sterility of drones (default parameters from Table S8, except *h* = 0).

*Case 2: Fitness neutral gene drive*

Next, we consider a gene drive (*h* > 0) with no effect on drone fertility (*p* = 0). This gives an indication of the potential for a driven gene to infiltrate a haplo-diploid system. Since the drive has no effect on fertility (*f* = 1) there is no impact on total population size and the outcome can be assessed by the dynamics of the wild-type queens. With perfect homing (*h* = 1),

*Q_ww_w_*• = *λZ^b^ g j* *Q_ww_w_*

Since *λZ^b^ g* = 1 when the population is at its equilibrium *K*, and *j* < 1 when carrier drones are present, WT queens *Q_ww_w_* must decrease in each subsequent generation and the driven gene will eventually infiltrate the whole population (Figure S12). The lower the homing rate *h*, the slower the spread of the modified gene. The effects of homing rate *h* and carrier drone competitiveness *c* on the rate of gene spread is shown in Figure S13.


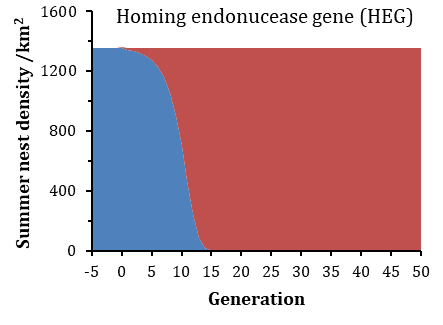


**Figure S12**. The effect of introducing 10 *Q_ii_w_* carrier queens /km^2^ into a wasp population at generation 0 with a HEG drive with no effect on carrier fitness (default parameters from Table S8, except *p* = 0).


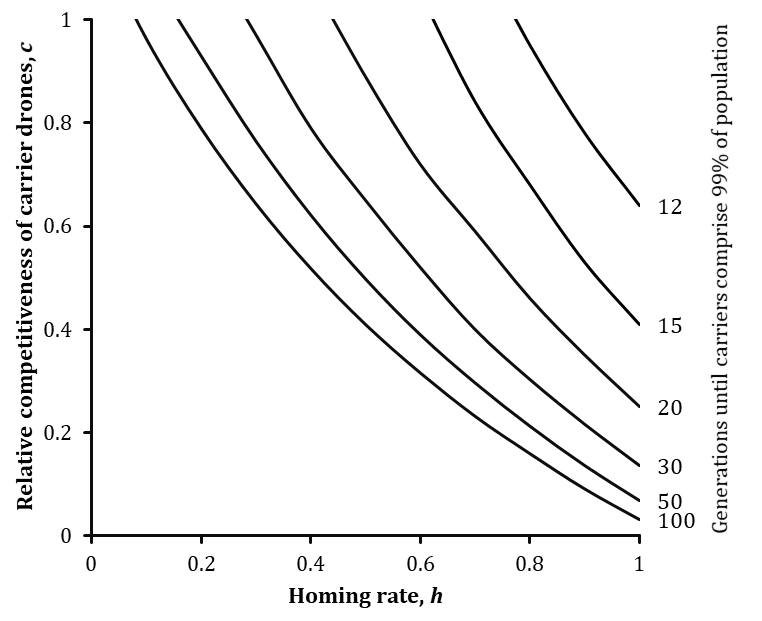


**Figure S13**. The effect of homing rate *h* and carrier drone competitiveness *c* on the number of generations required for carrier queens to comprise 99% of the population, following introduction of 100 carrier queens *Q_ii_w_* into a wasp population at its carrying capacity *K*.

*Case 3: Gene drive causing complete drone sterility*

We now consider scenarios involving a gene drive (*h* > 0) causing drone sterility (*p* > 0). We begin by examining the best-case scenario for wasp control: a drive with perfect homing (*h* = 1) that causes complete drone sterility (*p* = 1). This corresponds to the default parameter values in Table S8 and forms a baseline for considering feasibility of wasp control via gene-driven drone sterility. Here, the model reduces to:

*Q_ww_w_*• = *λZ^b^* *f* *g* *Q_ww_w_*

*Q_ii_w_*• = *λZ^b^* *f* *g Q_ii_w_*

Perfect homing (*h* = 1) means there are no heterozygous carrier queens (*Q_wi_w_* = *Q_wi_i_* = 0), and complete sterility (*p* = 1, so *j* = 1) means there are no summer queens that mated with carrier drones (*Q_ww_i_* = *Q_wi_i_* = *Q_ii_i_* = 0). The rate of change of WT queens is the same as that for carrier queens, so the HEG has neutral stability in the population, neither spreading nor declining over time (Figure S14).


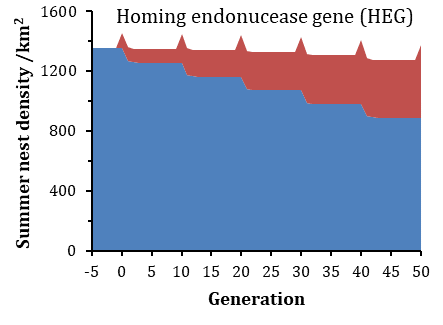


**Figure S14**. The effect of introducing 100 *Q_ii_w_* carrier queens /km^2^ into a wasp population at 10-yearly intervals with a perfect homing HEG causing complete sterility of carrier drones (default parameters from Table S8).

As more carriers are introduced into the population, the equilibrium population size begins to decline. The impact of carrier queens on population size depends on the number of matings per queen *m*, the sperm load fecundity factor *b*, and the relative competitiveness of carrier drones *c* (Figure S15). If carriers have no loss of mating competitiveness (*c* = 1), then the equilibrium population size may be estimated as

$$\frac{\sum Q}{K}=\frac{\lambda s\left( 1-x \right)^{b}\left( 1-x^{m} \right)-1}{\left( \lambda s-1 \right)\left( 1-x \right)^{b}}$$

where *x* = *Q_ii_w_*/(*Q_ww_w_* + *Q_ii_w_*) is the proportion of queens that are carriers.


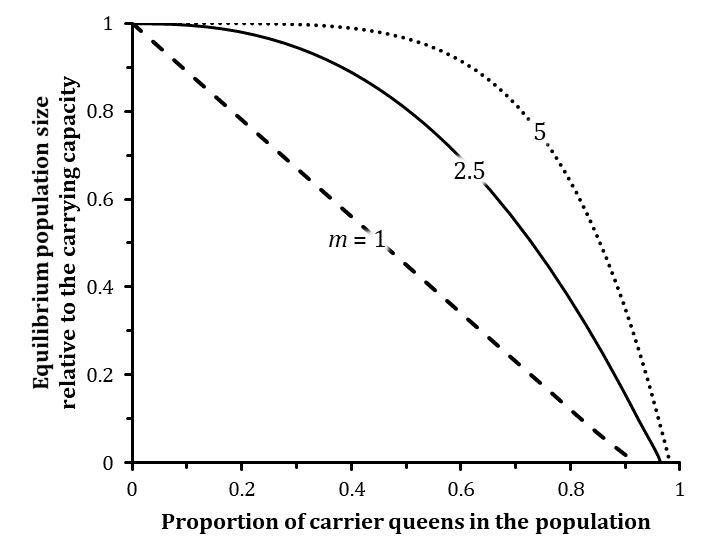

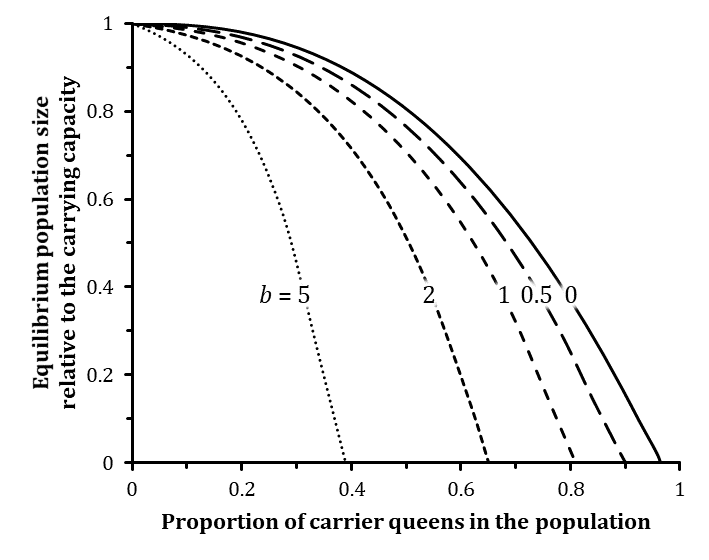

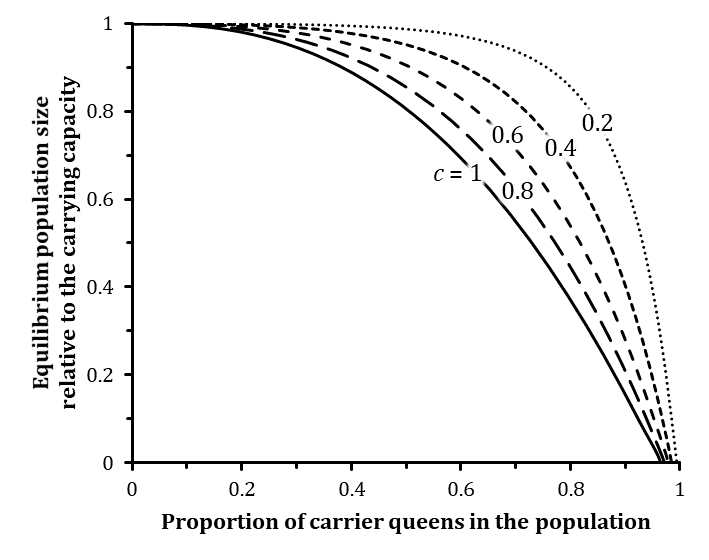


**Figure S15**. The effect of carrier queen prevalence on equilibrium population size, showing effects of the number of matings per queen *m* (top left), the sperm load fecundity factor *b*

(top right), and the relative competitiveness of carrier drones *c* (bottom). In each case the solid line corresponds to the default parameter set (Table S8).

For the default parameter set, reducing the population by half would require at least 72% of queens to carry the sterility gene, and population elimination would require more than 95% of queens to be carriers. Since the only way to increase carrier queens in the population is to release them, eradication would require rearing and releasing about 20 carrier queens for each wild queen. With typical densities of *K* = 1350 nests /km^2^, this seems infeasible. However, if releases were made immediately after the population was reduced by poison baiting then eradication is much more achievable. Intensive baiting can typically achieve around 95% reduction in New Zealand wasp populations ^25,26^. The model suggests that if 100 carrier queens /km^2^ were released immediately following a 95% population reduction then a single such operation might reduce wasp densities by 30%, and three operations may be sufficient to cause extinction (Figures S16 and S17).


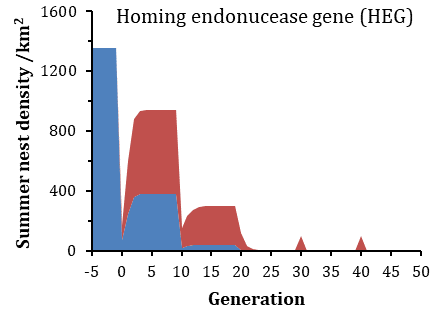


**Figure S16**. The effect of reducing a wasp population by 95% and introducing 100 carrier queens /km^2^ once every ten years. The gene drive has perfect homing and confers complete sterility of drones.


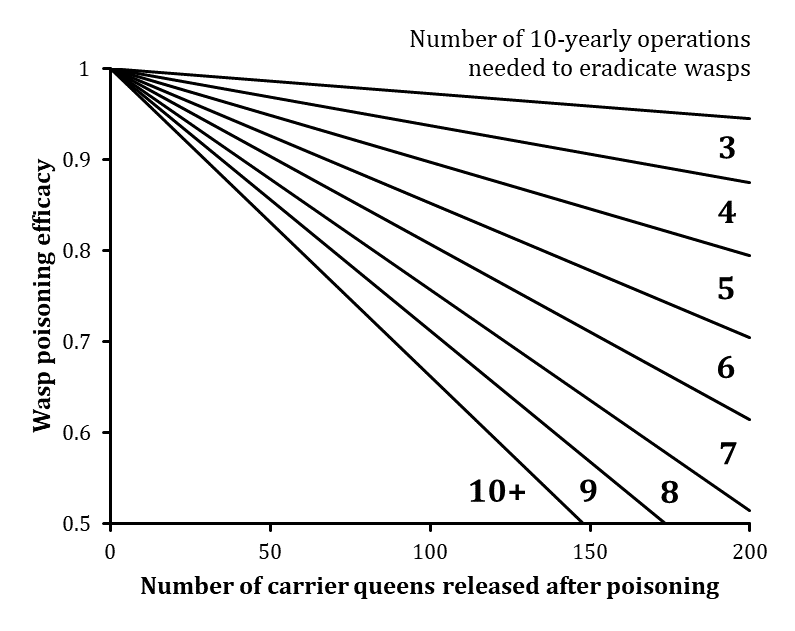


**Figure S17**. The effort required to eliminate a wasp population by a combination of poisoning (y axis) and release of *Q_ii_w_* females carrying a perfect homing HEG for complete drone sterility (x axis) with introductions every ten years.

If we relax the assumption of perfect homing the prospects for population control are less promising (Figure S18). With even *h* = 0.95 eradication is no longer possible with a combination of poisoning and a gene drive causing complete drone sterility.


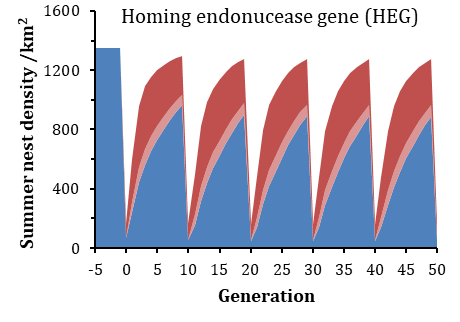


**Figure S18**. As for Figure S16 except that the gene drive has 80% homing.

*Case 4: Gene drive causing partial drone sterility*

Now we consider a gene drive causing sterility in a proportion *p* of carrier drones while the rest remain fertile. This is functionally equivalent to a proportion *p* of matings involving any carrier drone being unsuccessful at transferring sperm. This may result in substantial population suppression (Figure S19).


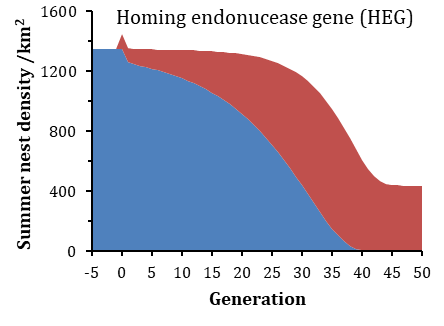


**Figure S19**. The effect of a gene drive causing sterility of *p* = 70% of carrier drones, introduced as 100 carrier queens at generation 0.

Population size equilibrates once the modified gene has been driven through the population so that all queens are *Q_ii_i_* and *Z* = 1 – *p*, *f* = 1 – *p^m^* and *j* = 0. The model reduces to

*Q_ii_i_*• = *λ*(1 – *p*)*^b^*^+1^ (1 – *p^m^*) *g* *Q_ii_i_*

At equilibrium, *Q_ii_i_*• = *Q_ii_i_*, and a little algebra shows that the relative population size is

$$\frac{Q_{ii\_i}}{K}=\frac{\lambda s\left( 1-p \right)^{b+1}\left( 1-p^{m} \right)-1}{\left( \lambda s-1 \right)\left( 1-p \right)^{b+1}}$$

Figure S20 shows the relative population sizes resulting from different combinations of *b* and *p*. Both the sperm load fecundity factor and the proportion of drones that are sterile increase population suppression and may cause extinction once the gene has spread through the population. For the default wasp parameters (Table S8) eradication might be achieved when *p* ≥ 0.8, so that at least 80% of carrier drones are sterile.


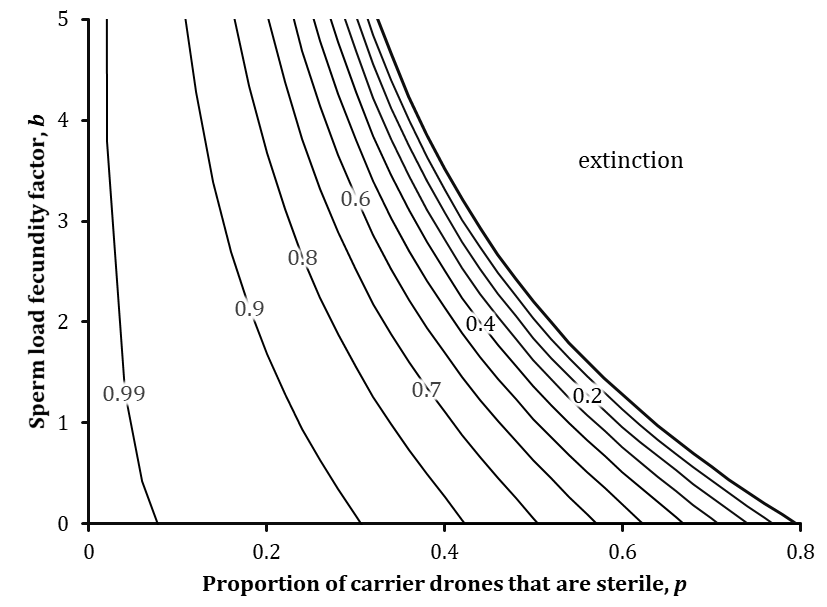


**Figure S20**. Relative size of wasp equilibrium populations resulting after full infiltration of a gene drive causing a proportion *p* of drones to be sterile and with queen fecundity dependent on sperm load according to factor *b*.

This result relies on the gene infiltrating the population, but as in the previous case gene spread is constrained by its effect on drone fertility. If relatively few carrier drones are sterilised then the gene will spread rapidly but will not have much effect on population density. Conversely, a gene that sterilises most carriers may suppress the population but will take a long time to spread through it. The effect on population size is independent of the homing rate *h* (see previous equation), but homing does influence the spread rate. Figure S21 summarises the constraints on population suppression imposed by the homing rate.


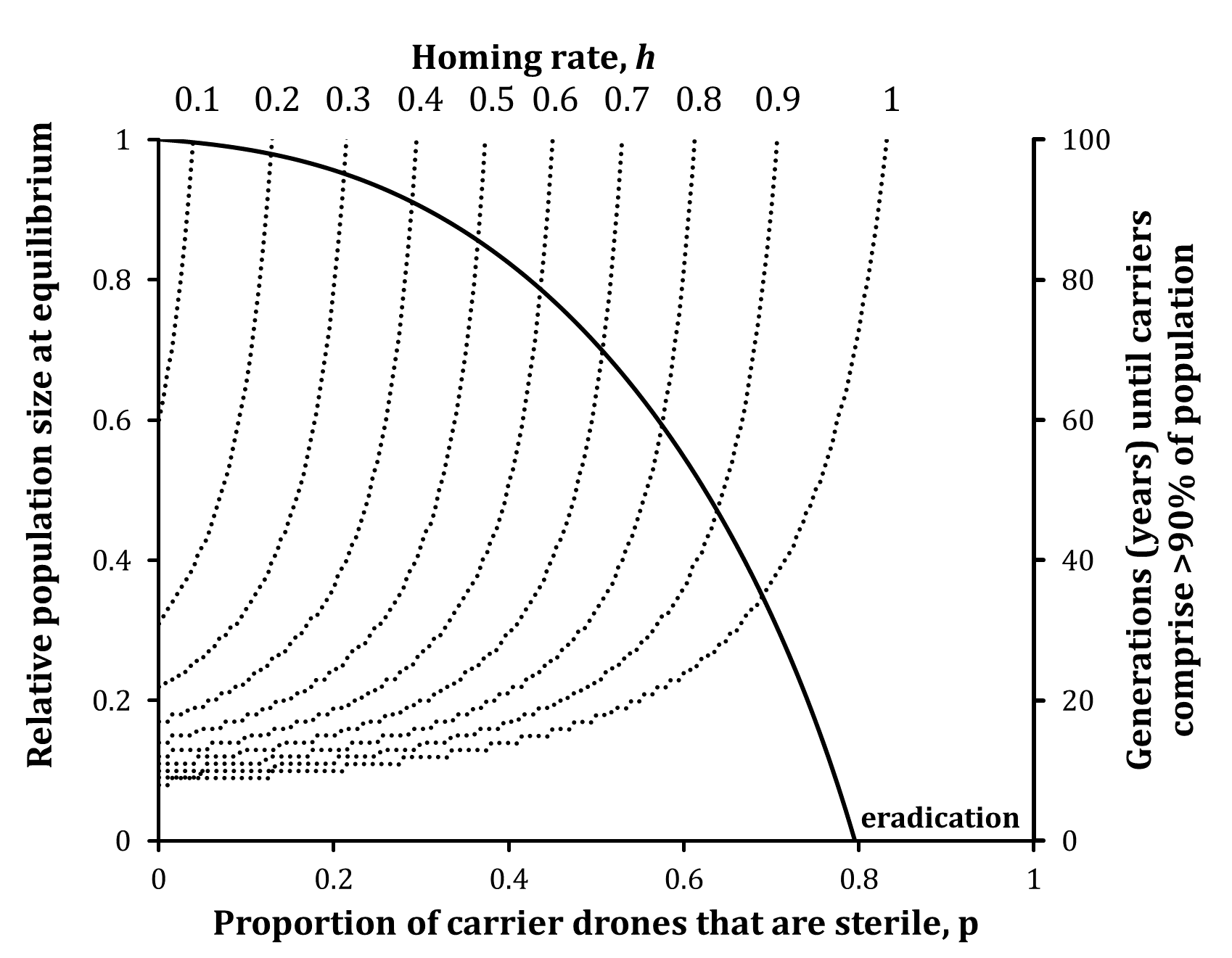


**Figure S21**. The potential population suppression (solid line, left axis) caused by a gene drive for partial drone sterility is not affected by the homing rate. However, the time taken for the drive to spread through >90% of the population (dotted lines, right axis) is determined largely by the homing rate, *h*. Thus, eradication is possible when *p* > 0.8, but only if the homing rate is sufficiently high to allow the drive to spread (*h* > 0.92). Even with perfect homing (*h* = 1) spread and subsequent eradication is predicted to require about a century.

Finally, we examined results from a stochastic integer-based version of the model, with reproductive processes modelled as draws from a Poisson distribution and proportions as draws from a binomial distribution. We were also careful to ensure conservation of mass. This effectively adds demographic stochasticity and gene drift to the model, but did not alter the overall patterns observed (e.g. Figure S22).


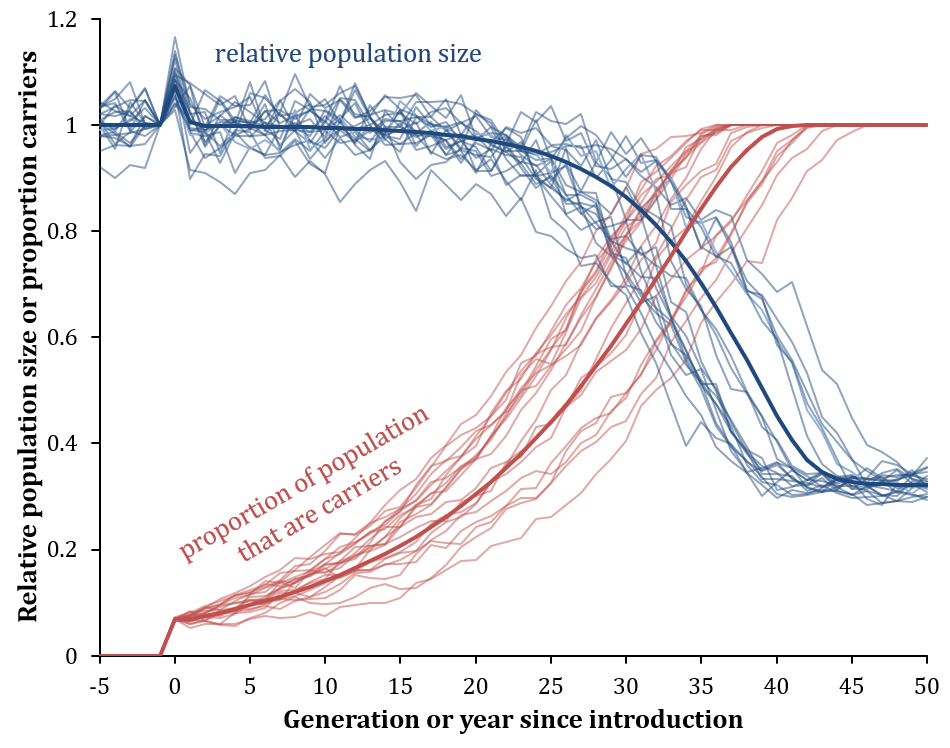


**Figure S22**. Results from 20 stochastic simulations of the scenario shown in Figure S19, with the deterministic results overlaid as a heavier line.

**Fig. S23**. Quantification of sgRNA-Cas9 cleavage assays. Selected samples were amplified by PCR and then digested with sgRNA-Cas9 (+), or with Cas9 alone (-), followed by agarose gel electrophoresis: (a) *Boule* v1 sgRNA; (b) *sdic* v1 sgRNA; (c) *sdic* v2 sgRNA; (d) *ocnus* v1 sgRNA; (e) *ocnus* v2 sgRNA. The sample numbers on the gel correspond to the Gel ID numbers in Supplementary Table 7, and EU/NZ indicates the samples were collected from either Europe or New Zealand. (f) The densities of the product bands were measured using Fiji image analysis software ^27^, and used to calculate average cleavage (expressed as the percentage of Cas9-alone treated PCR products) and standard error (based on the standard deviation).

# **
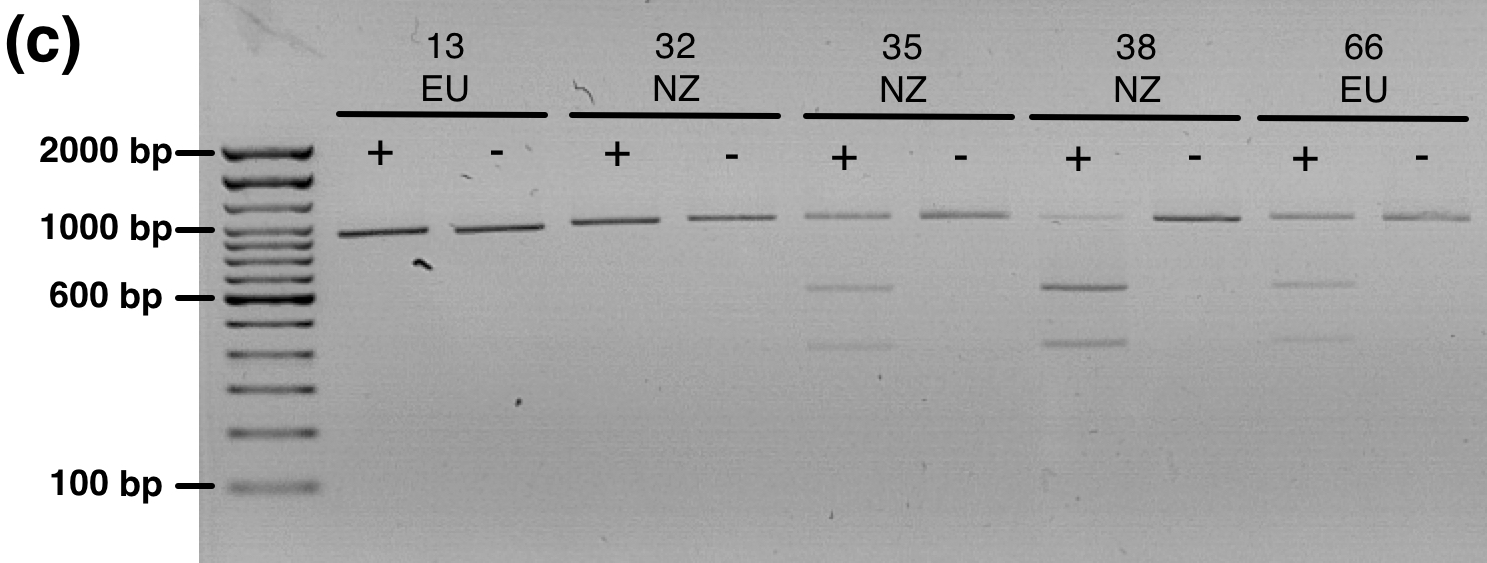

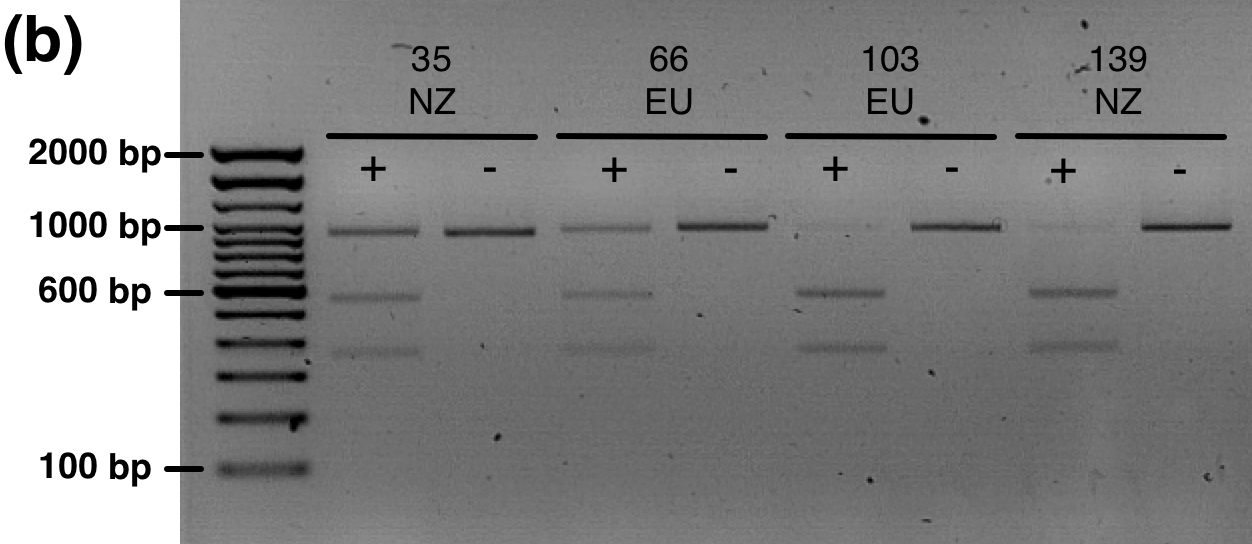
**
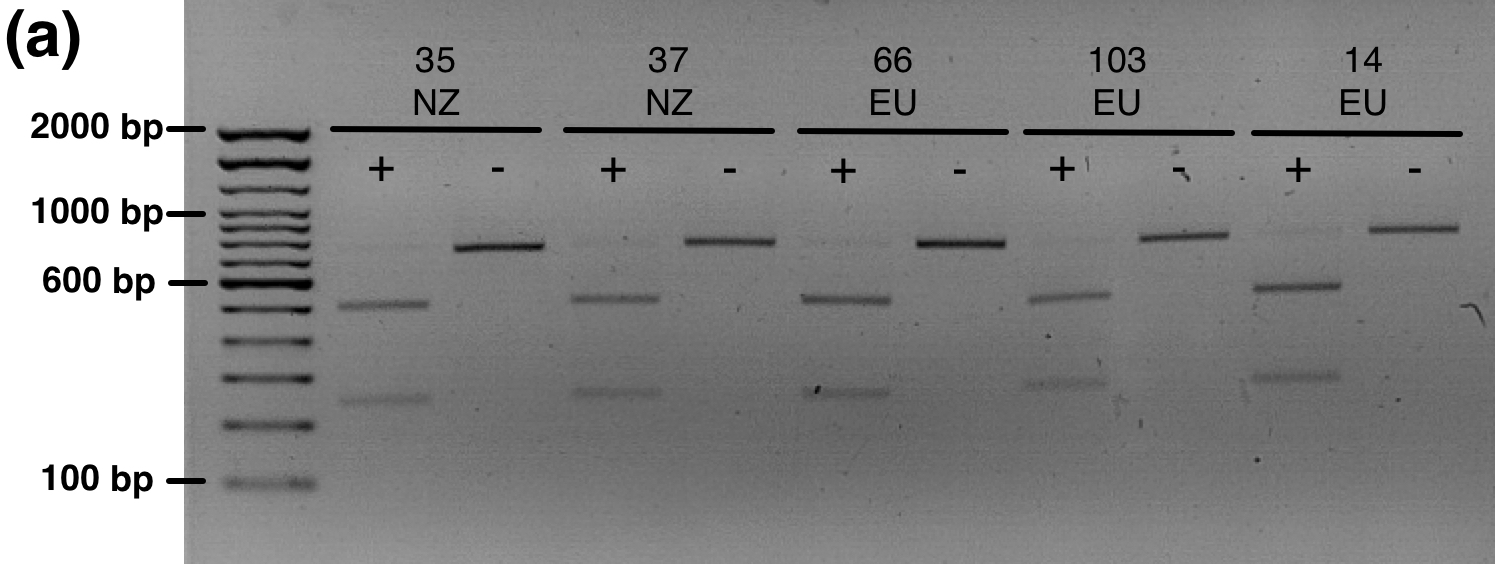

**
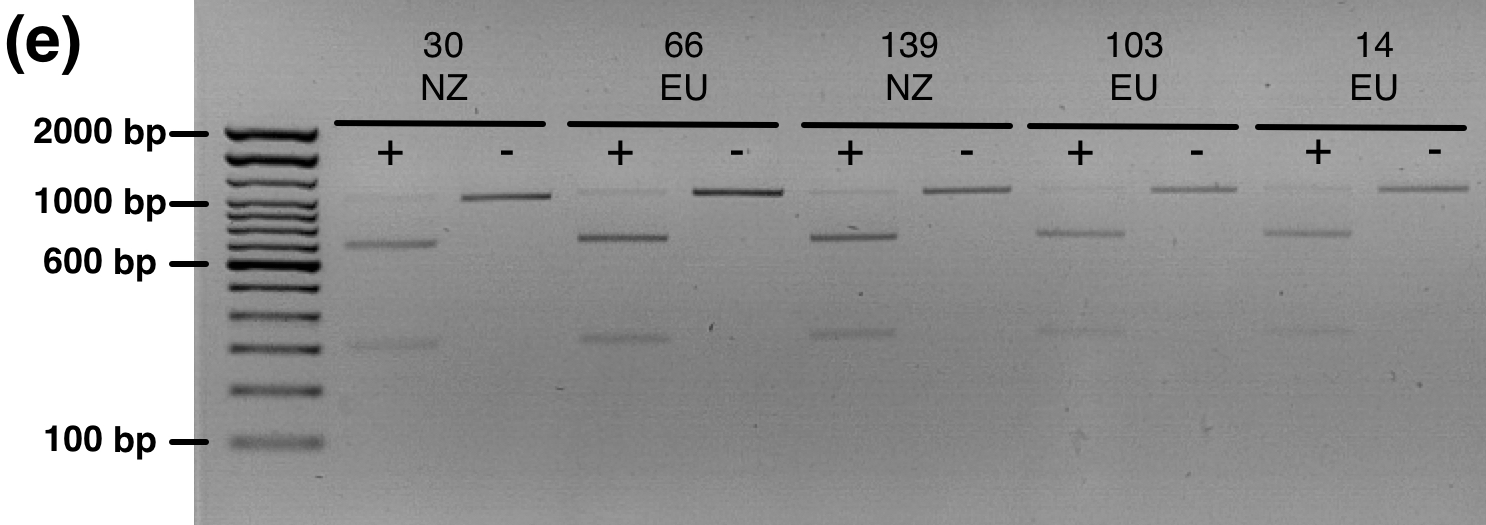

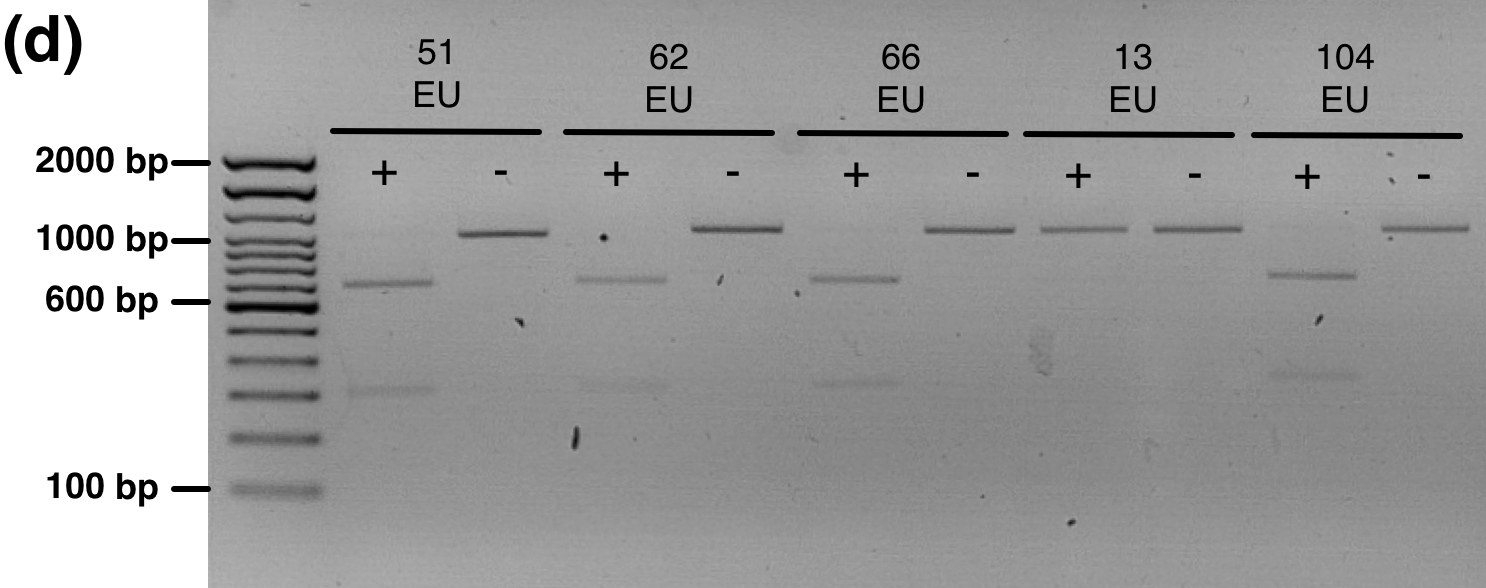
**

# **Glossary**

**carrier**: individual carrying the modified gene of interest

**diploid**: mating system in which both males and females have two complete sets of chromosomes, and where sex is typically determined by variant X and Y chromosomes

**drone**: haploid male wasp

**effective sperm load**: the ratio of fertile sperm to total sperm collected at mating

**fertilization rate**: the proportion gynes receiving viable sperm over all matings

**gyne**: a young queen wasp in autumn/winter

**haplo-diploid**: mating system in which males are haploid with one complete set of chromosomes, while females are diploid with two complete sets of chromosomes

**homing rate**: the strength of the gene drive: the proportion of heterozygous genetic modification carriers that are rendered homozygous for the gene drive

**queen**: diploid female breeding wasp

**survival factor**: proportion of gynes successfully overwintering and securing nest sites in spring

**Trojan female**: a female carrying a cytoplasmic gene that reduces the fertility of her male offspring

**vespulid**: belonging to the wasp genus *Vespula*

**worker**: non-breeding diploid wasp

**WT**: carrying only the unmodified form (“wild type”) of the gene of interest

**SI References**

1 Emms, D. M. & Kelly, S. OrthoFinder: solving fundamental biases in whole genome comparisons dramatically improves orthogroup inference accuracy. *Genome Biol.* **16**, 157, doi:10.1186/s13059-015-0721-2 (2015).

2 Elsik, C. G. *et al.* Hymenoptera Genome Database: integrating genome annotations in HymenopteraMine. *Nucleic Acids Res.* **44**, D793-800, doi:10.1093/nar/gkv1208 (2016).

3 Nygaard, S. *et al.* The genome of the leaf-cutting ant *Acromyrmex echinatior* suggests key adaptations to advanced social life and fungus farming. *Genome Res.* **21**, 1339-1348, doi:10.1101/gr.121392.111 (2011).

4 Elsik, C. G. *et al.* Finding the missing honey bee genes: lessons learned from a genome upgrade. *BMC Genomics* **15**, 86, doi:10.1186/1471-2164-15-86 (2014).

5 Suen, G. *et al.* The genome sequence of the leaf-cutter ant *Atta cephalotes* reveals insights into its obligate symbiotic lifestyle. *PLoS Genet.* **7**, e1002007, doi:10.1371/journal.pgen.1002007 (2011).

6 Sadd, B. M. *et al.* The genomes of two key bumblebee species with primitive eusocial organization. *Genome Biol.* **16**, 76, doi:10.1186/s13059-015-0623-3 (2015).

7 Gupta, S. K. *et al.* Scrutinizing the immune defence inventory of *Camponotus floridanus* applying total transcriptome sequencing. *BMC Genomics* **16**, 540, doi:10.1186/s12864-015-1748-1 (2015).

8 Oxley, P. R. *et al.* The genome of the clonal raider ant *Cerapachys biroi*. *Curr. Biol.* **24**, 451-458, doi:10.1016/j.cub.2014.01.018 (2014).

9 Kocher, S. D. *et al.* The draft genome of a socially polymorphic halictid bee, *Lasioglossum albipes*. *Genome Biol.* **14**, R142 (2013).

10 Smith, C. D. *et al.* Draft genome of the globally widespread and invasive Argentine ant (*Linepithema humile*). *Proc. Natl. Acad. Sci. U.S.A.* **108**, 5673-5678, doi:10.1073/pnas.1008617108 (2011).

11 Werren, J. H. *et al.* Functional and evolutionary insights from the genomes of three parasitoid *Nasonia* species. *Science* **327**, 343-348, doi:10.1126/science.1178028 (2010).

12 Smith, C. R. *et al.* Draft genome of the red harvester ant *Pogonomyrmex barbatus*. *Proc Natl Acad Sci U S A* **108**, 5667-5672, doi:10.1073/pnas.1007901108 (2011).

13 Wurm, Y. *et al.* The genome of the fire ant *Solenopsis invicta*. *Proc. Natl. Acad. Sci. U.S.A.* **108**, 5679-5684, doi:10.1073/pnas.1009690108 (2011).

14 Stover, B. C. & Muller, K. F. TreeGraph 2: combining and visualizing evidence from different phylogenetic analyses. *BMC Bioinformatics* **11**, 7, doi:10.1186/1471-2105-11-7 (2010).

15 Thomas, P. D. *et al.* PANTHER: a library of protein families and subfamilies indexed by function. *Genome Res.* **13**, 2129-2141, doi:10.1101/gr.772403 (2003).

16 Stein, K. J. & Fell, R. D. Correlation of queen sperm content with colony size in yellowjackets (Hymenoptera: Vespidae). *Environ. Entomol.* **23**, 1497-1500, doi:DOI 10.1093/ee/23.6.1497 (1994).

17 Goodisman, M. A., Matthews, R. W. & Crozier, R. H. Mating and reproduction in the wasp *Vespula germanica*. *Behav. Ecol. Sociobiol.* **51**, 497-502, doi:10.1007/s00265-002-0470-6 (2002).

18 Dobelmann, J. *et al.* Fitness in invasive social wasps: the role of variation in viral load, immune response and paternity in predicting nest size and reproductive output. *Oikos* **126**, 1208-1218, doi:10.1111/oik.04117 (2017).

19 Masciocchi, M., Martinez, A. S., Pereira, A. J., Villacide, J. M. & Corley, J. C. Dispersal behavior of yellowjacket (*Vespula germanica*) queens. *Insect Sci.* **25**, 109-116, doi:10.1111/1744-7917.12374 (2018).

20 Crozier, R. H. & Bruckner, D. Sperm clumping and the population genetics of Hymenoptera. *Am. Nat.* **117**, 561-563 (1981).

21 Barlow, N. D., Beggs, J. R. & Barron, M. C. Dynamics of common wasps in New Zealand beech forests: a model with density dependence and weather. *J. Appl. Ecol.* **71**, 663-671 (2002).

22 Lester, P. J., Haywood, J., Archer, M. E. & Shortall, C. R. The long-term population dynamics of common wasps in their native and invaded range. *J. Anim. Ecol.* **86**, 337-347, doi:10.1111/1365-2656.12622 (2017).

23 Gruber, M. A. M. *et al.* Fitness and microbial networks of the common wasp, *Vespula vulgaris* (Hymenoptera: Vespidae), in its native and introduced ranges. *Ecol. Entomol.* **44**, 512-523, doi:10.1111/een.12732 (2019).

24 Archer, M. E. Population dynamics of the social wasps *Vespula vulgaris* and *Vespula germanica* in England. *J. Anim. Ecol.* **54**, 473-485 (1985).

25 Edwards, E., Toft, R., Joice, N. & Westbrooke, I. The efficacy of Vespex® wasp bait to control *Vespula* species (Hymenoptera: Vespidae) in New Zealand. *Int. J. Pest Manage.* **63**, 266-272, doi:10.1080/09670874.2017.1308581 (2017).

26 Harris, R. J. & Etheridge, N. D. Comparison of baits containing fipronil and sulfluramid for the control of *Vespula* wasps. *New Zeal. J. Zool.* **28**, 39-48, doi:10.1080/03014223.2001.9518255 (2001).

27 Schindelin, J. *et al.* Fiji: an open-source platform for biological-image analysis. *Nat. Methods* **9**, 676-682, doi:10.1038/nmeth.2019 (2012).
